# Mitochondrial DNA Mutations Induce Mitochondrial Dysfunction, Apoptosis and Sarcopenia in Skeletal Muscle of Mitochondrial DNA Mutator Mice

**DOI:** 10.1371/journal.pone.0011468

**Published:** 2010-07-07

**Authors:** Asimina Hiona, Alberto Sanz, Gregory C. Kujoth, Reinald Pamplona, Arnold Y. Seo, Tim Hofer, Shinichi Someya, Takuya Miyakawa, Chie Nakayama, Alejandro K. Samhan-Arias, Stephane Servais, Jamie L. Barger, Manuel Portero-Otín, Masaru Tanokura, Tomas A. Prolla, Christiaan Leeuwenburgh

**Affiliations:** 1 Division of Biology of Aging, Department of Aging and Geriatric Research, Institute on Aging, College of Medicine, University of Florida, Gainesville, Florida, United States of America; 2 Mitochondrial Gene Expression and Disease Group. Institute of Medical Technology and Tampere University Hospital, University of Tampere, Tampere, Finland; 3 Department of Genetics and Medical Genetics, University of Wisconsin, Madison, Wisconsin, United States of America; 4 Department of Experimental Medicine, University of Lleida-Institut de Recerca Biomèdica de Lleida, Lleida, Spain; 5 Department of Applied Biological Chemistry, The University of Tokyo, Bunkyo-ku, Tokyo, Japan; 6 Department of Biochemistry and Molecular Biology, Faculty of Sciences, University of Extremadura, Badajoz, Spain; 7 LifeGen Technologies, LLC, Madison, Wisconsin, United States of America; McMaster University, Canada

## Abstract

**Background:**

Aging results in a progressive loss of skeletal muscle, a condition known as sarcopenia. Mitochondrial DNA (mtDNA) mutations accumulate with aging in skeletal muscle and correlate with muscle loss, although no causal relationship has been established.

**Methodology/Principal Findings:**

We investigated the relationship between mtDNA mutations and sarcopenia at the gene expression and biochemical levels using a mouse model that expresses a proofreading-deficient version (D257A) of the mitochondrial DNA Polymerase γ, resulting in increased spontaneous mtDNA mutation rates. Gene expression profiling of D257A mice followed by Parametric Analysis of Gene Set Enrichment (PAGE) indicates that the D257A mutation is associated with a profound downregulation of gene sets associated with mitochondrial function. At the biochemical level, sarcopenia in D257A mice is associated with a marked reduction (35–50%) in the content of electron transport chain (ETC) complexes I, III and IV, all of which are partly encoded by mtDNA. D257A mice display impaired mitochondrial bioenergetics associated with compromised state-3 respiration, lower ATP content and a resulting decrease in mitochondrial membrane potential (Δψ_m_). Surprisingly, mitochondrial dysfunction was not accompanied by an increase in mitochondrial reactive oxygen species (ROS) production or oxidative damage.

**Conclusions/Significance:**

These findings demonstrate that mutations in mtDNA can be causal in sarcopenia by affecting the assembly of functional ETC complexes, the lack of which provokes a decrease in oxidative phosphorylation, without an increase in oxidative stress, and ultimately, skeletal muscle apoptosis and sarcopenia.

## Introduction

The progressive loss of skeletal muscle mass and strength observed in older individuals is a condition known as sarcopenia. The associated muscle atrophy and weakness directly affect the functional capacity and quality of life of the elderly. Sarcopenia affects a growing population, occurring in 10–25% of individuals under the age of 70 and in more than 40% of the elderly over the age of 80 [1], [2]. The annual cost of treating sarcopenia is greater than the amount spent due to osteoporosis, yet little effort is made to increase public awareness to prevent sarcopenia [3].

Despite the fact that in animal cells mtDNA comprises only 1–3% of genetic material, several lines of evidence suggest that its contribution to cellular physiology could be much greater than would be expected from its size alone [4], [5]. For instance, it mutates at higher rates than nuclear DNA [6], [7], which is probably a consequence of: (a) its close proximity to the ETC, and thus of greater exposure to reactants, (b) its lack of protective histones and introns, and (c) its lack of extensive DNA repair systems compared to the nuclear DNA [8], [9], [10]. Importantly, defects in the ETC can have pleiotropic effects because they affect cellular energetics as a whole [4]. For these reasons, a central role for mitochondrial DNA (mtDNA) mutations in aging has been postulated [11], [12], [13]. Indeed, mtDNA mutations have been shown to accumulate with aging in several tissues, including skeletal muscle of various species [14], [15], [16], [17], [18], [19].

Previous studies have provided strong, though correlative, experimental support for an association between mtDNA mutations and tissue dysfunction, particularly in long-lived post-mitotic cells such as cardiomyocytes, skeletal muscle fibers and neurons [14], [16], [17], [18], [19]. In order to determine whether mtDNA mutations underlie sarcopenia, we used a genetically engineered mouse model that expresses a proofreading-deficient version of the mitochondrial DNA polymerase gamma (D257A), resulting in increased spontaneous mutation rates in mtDNA [20], [21]. We have previously characterized accelerated aging in D257A mice [20] and found that these mice exhibited multiple age-related phenotypes including thymic involution, loss of bone mass, cardiac dysfunction and hearing loss. These findings provide support for the hypothesis that mtDNA mutations can play a causal role in mammalian aging [20], [21]. Therefore, the D257A mouse may provide an *in vivo* model to study the mechanisms of skeletal muscle loss with age, specifically, the contribution of mtDNA mutations.

The purpose of the present study was to identify the specific molecular mechanism that links mtDNA mutations and the loss of skeletal muscle mass with age in mitochondrial mutator mice [20]. The central hypothesis tested was that mutations in mitochondrial DNA, known to be associated with aging in many post-mitotic tissues, play a causal role in skeletal muscle loss of mutator mice by affecting the assembly of fully functional ETC complexes, leading to mitochondrial dysfunction and ultimately, to the activation of a mitochondrial-mediated apoptotic program.

## Results

### D257A mice display significant skeletal muscle loss

**Figure 1:**
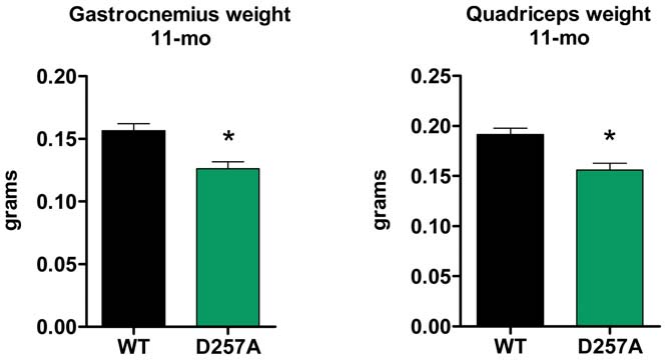
D257A mice display significant skeletal muscle loss. At 11 mo of age, gastrocnemius (n = 22 per group, *p<0.001) and quadriceps (n = 22 per group, *p<0.001) mean muscle mass in D257A mice is significantly decreased compared to age-matched WT. Error bars represent SEM.

At 11-mo of age, D257A mice exhibited significant skeletal muscle loss of gastrocnemius (wild-type, WT: 0.157±0.006 vs. D257A: 0.126±0.006 grams, p = 0.0004, −20%) and of quadriceps muscle (WT: 0.190±0.006 vs. D257A: 0.156±0.007 grams, p = 0.0003, −18% ) compared to WT mice (Fig. 1). The level of sarcopenia is similar to that observed in gastrocnemius of normally-aged (30-mo WT) mice compared to young mice (5-mo WT) (gastrocnemius, 5-mo: 0.146±0.009 vs. 30-mo: 0.110±0.007 grams, p = 0.0095, −24.5%), but less than the level of sarcopenia observed in quadriceps of aged WT mice (5-mo: 0.177±0.004 vs. 30-mo: 0.105±0.007 grams, p = 0.0095, −40%). We next examined whether the observed sarcopenia was due primarily to a reduction in muscle fiber size or fiber number and whether type I or type II fibers were differentially affected. Muscle fiber number and diameter were determined as described [22] for gastrocnemius muscles isolated from 3-mo and 13-mo WT and D257A mice. There were no significant differences between genotypes in the number of type I or type II fibers at either age, although there was a trend toward a lower proportion of type I fibers in the gastrocnemius of D257A mice at both ages (Table 1). By contrast, we observed a significant decrease in the diameter of type I (but not type II) fibers at 13 months of age.

**Table 1:** Fiber type analysis in the gastrocnemius muscle of wild-type and D257A mice.

Age	Genotype	Type I Fiber diameter (µm, mean ± SEM)	Type I Fiber number (mean ± SEM)	Type I Proportion (%, mean ± SEM)	Type II Fiber diameter (µm, mean ± SEM)	Type II Fiber number (mean ± SEM)	Type II Proportion (%, mean ± SEM)
3 mo	WT	22.985±0.258	1793±85	27.257±1.330	31.320±0.303	4832±351	72.743±1.330
3 mo	D257A	22.816±0.610	1511±128	22.659±1.427	33.072±0.980	5144±233	77.341±1.427
13 mo	WT	25.494±1.368	1533±127	26.584±1.051	31.938±1.373	4203±188	73.416±1.051
13 mo	D257A	20.535±0.516*	1612±62	24.792±1.265	28.930±1.525	4929±271	75.208±1.265

* p<0.01, 13-month WT vs D257A type I fiber diameter comparison. All other comparisons, p>0.05.

### Gene expression profiling of D257A mice

**Figure 2:**
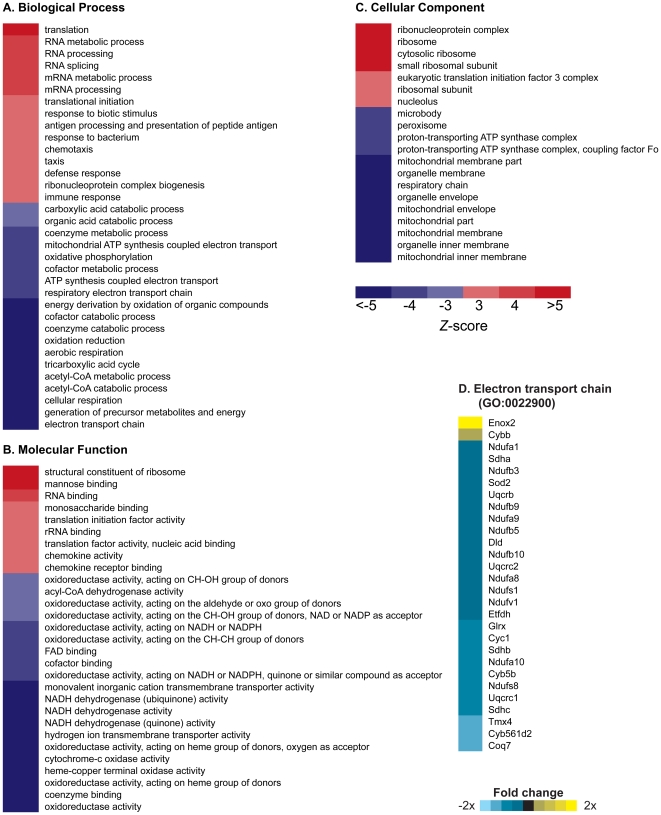
Parametric Analysis of Gene Set Enrichment identifies Gene Ontology (GO) terms changed in gastrocnemius muscle of 13-month old D257A mice as compared to 13-month old WT mice. (A, B, C) Terms shown are those significantly changed (*p*<0.01, FDR<0.01). Red indicates a GO term that was induced by mtDNA mutations (*Z*-score>0); blue indicates GO terms that were suppressed by mtDNA mutations (*Z*-score<0). n = 5 mice per group. (D) Genes listed within the GO term “electron transport chain” (*Z*-score = −8.89; p<0.0001) whose expression level significantly (p<0.01) increased (yellow) or decreased (blue) in D257A mice.

Affymetrix Mouse Genome 430 2.0 arrays were used to measure gene expression profiles in gastrocnemius muscle of 13-month old WT and D257A mice. We then performed Parametric Analysis of Gene Set Enrichment (PAGE) [23] to identify classes of genes that were differentially expressed as a result of mtDNA mutations induced by the error-prone mtDNA Polymerase γ. Comparison of 13-month old D257A mice with 13-month old WT mice revealed numerous gene set categories significantly changed in expression (Fig. 2A–C). D257A mice were associated with a dramatic downregulation of “Biological Process” gene sets associated with mitochondrial function, including *electron transport chain* (GO:0022900), *aerobic respiration* (GO:0009060), and *generation of precursor metabolites and energy* (GO:0006091) (Fig. 2A). Downregulation of “Molecular Function” gene sets was also associated with decreased mitochondrial function, including *oxidoreductase activity* (GO:0016491), *NADH dehydrogenase activity* (GO:0050662) and *cytochrome c oxidase activity* (GO:0004129) (Fig. 2B). In the broader “Cellular Component” gene set category, the gene sets *mitochondrion* (GO:0005739), *mitochondrial membrane* (GO:0031966) and *mitochondrial inner membrane* (GO:0005739) were downregulated (Fig. 2C). These observations suggest that there is a transcriptional program to reduce the expression of nuclear-encoded, mitochondrial transcripts in cells that carry functionally impaired mitochondria that result from the accumulation of mtDNA mutations.

Interestingly, gene sets associated with ribosomes or RNA synthesis were the only category of gene sets upregulated in D257A mice. Possibly, impaired bioenergetics in these animals is associated with alterations in protein synthesis and a resulting attempt to increase protein synthesis through overexpression of ribosomal/translation-related genes. Remarkably, no gene sets associated with apoptosis or DNA damage were induced, suggesting that apoptosis in D257A mice may be an intrinsic mitochondrial process that occurs in the absence of transcriptional induction of classical modulators of mitochondrial apoptosis in response to DNA damage, such as Bak and Bax [24]. This observation is in stark contrast with our previous DNA microarray analysis of skeletal muscle of aged WT animals, which is associated with a marked induction of genes associated with a p53-mediated apoptotic response [25].

At the individual gene expression level, 97 genes were significantly changed in expression in the 13-month old WT vs D257A comparison (*p*<0.05, FDR <0.05). Mitochondrial Creatine Kinase 2 (*Ckmt2*) was the most significantly altered mitochondrial function transcript (fold change  = 1.7, *p* = 10^−7^, FDR  = 0.004) (Table S1). Interestingly, comparison between 3-month old WT and 3-month old D257A mice under the same statistical parameters (including same FDR stringency) revealed no significant changes in gene expression either at the individual gene, or PAGE analysis. Thus, changes in gene expression due to the D257A mutation in skeletal muscle develop later in life in D257A mice, possibly as a consequence of progressive mtDNA mutation accumulation and associated mitochondrial dysfunction.

### D257A mice display decreased content and gene expression of ETC Complexes I, III, and IV

**Figure 3:**
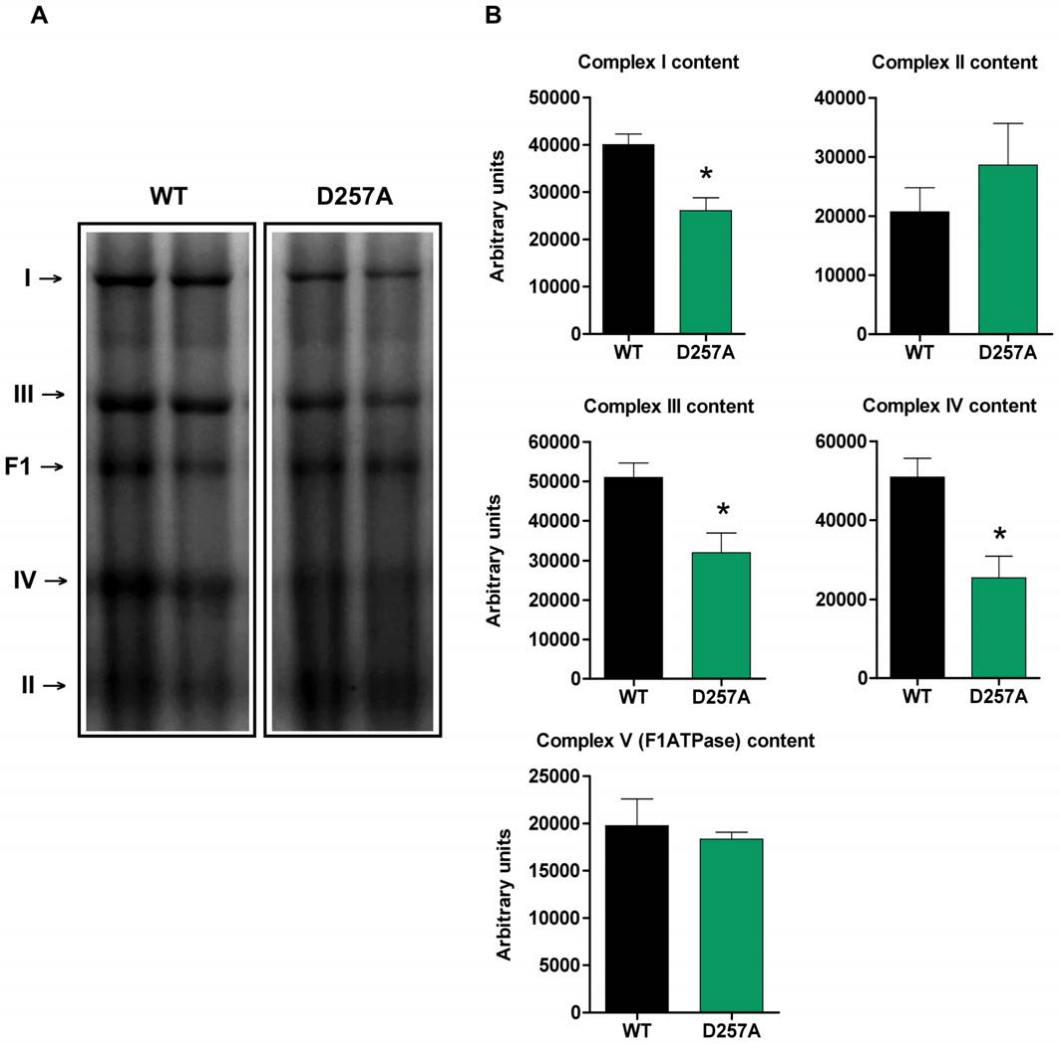
D257A mice display decreased content of ETC Complexes I, III and IV compared to WT. (A) The total content of ETC complexes I, II, III, IV and the F1 domain of the ATPase from skeletal muscle of 11-mo old WT and D257A mice (n = 7 per group) was determined using Blue Native PAGE electrophoresis followed by staining with commassie blue stain. Proteins were separated according to molecular weight. Representative blots are depicted. (B) Statistical analysis of ETC complexes content. Arbitrary units represent densitometry values normalized to total protein loaded measured by the Bradford assay. Mean values ±SEM are shown. *p<0.01.

We measured the content of ETC complexes I, II, III, IV and the F1 domain of ATPase in 11-mo old WT and D257A skeletal muscle using blue native PAGE (Fig. 3). We found that the total content of complexes I (WT: 40050±2281 vs. D257A: 26100±2724 arbitrary units, p = 0.002, −35%), III (WT: 50970±3673 vs. D257A: 31960±4925 arbitrary units, p = 0.0093, −37%), and IV (WT: 50900±4782 vs. D257A: 25460±5532 arbitrary units, p = 0.0046, −50%), all of which contain subunits encoded by mtDNA, were profoundly reduced in D257A mice (Fig. 3A, 3B). In contrast, the content of complex II (WT: 20710±4079 vs. D257A: 28610±7051 arbitrary units, p = 0.3513) and ATPase F1 (WT: 19760±2831 vs. D257A: 18330±747.9 arbitrary units, p = 0.64), both of which contain only nuclear-encoded subunits, was not different between genotypes (Fig. 3A, 3B). This observation reinforces the idea that the accumulation of mtDNA point mutations directly affects the assembly of complexes that are partly mitochondrial-encoded [26], while strictly nuclear-encoded mitochondrial complexes appear unaffected.

**Figure 4:**
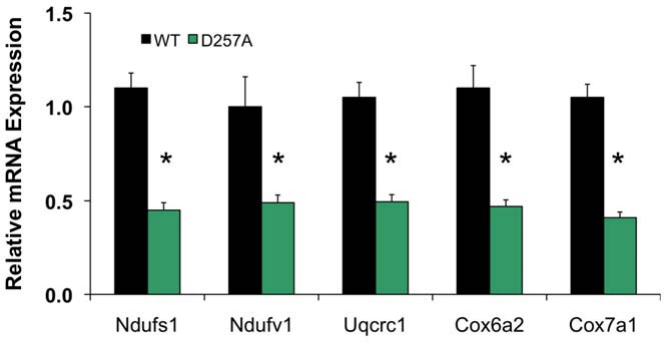
D257A mice display decreased mRNA expression of nuclear genes encoding components of ETC Complexes I, III and IV. Relative mRNA expression of genes encoding components of the ETC complexes I, III and IV, including *Ndufs1, Ndufv1, Uqcrc1, Cox6a2*, and *Cox7a1* was measured in the skeletal muscle from WT and D257A mice at 13 months of age by quantative RT-PCR (n = 5 per group). Mean values ±SEM are shown. *p<0.05.

We also measured relative mRNA levels of nuclear encoded genes of the ETC complexes I, III, and IV in 13-mo old WT and D257A skeletal muscle by quantitative RT-PCR (Fig. 4). We found that the mean relative mRNA expression of *Ndufs1 (complex I), Ndufv1 (complex I), Uqcrc1 (complex III), Cox6a2 (complex IV)*, and *Cox7a1 (complex IV)* in the skeletal muscle of D257A mice was significantly lower than that of WT mice (Fig. 4). These measurements are in agreement with the general finding of reduced expression of gene sets encoding mitochondrial proteins.

### ETC complexes specific activities are unaffected in D257A mice

**Figure 5:**
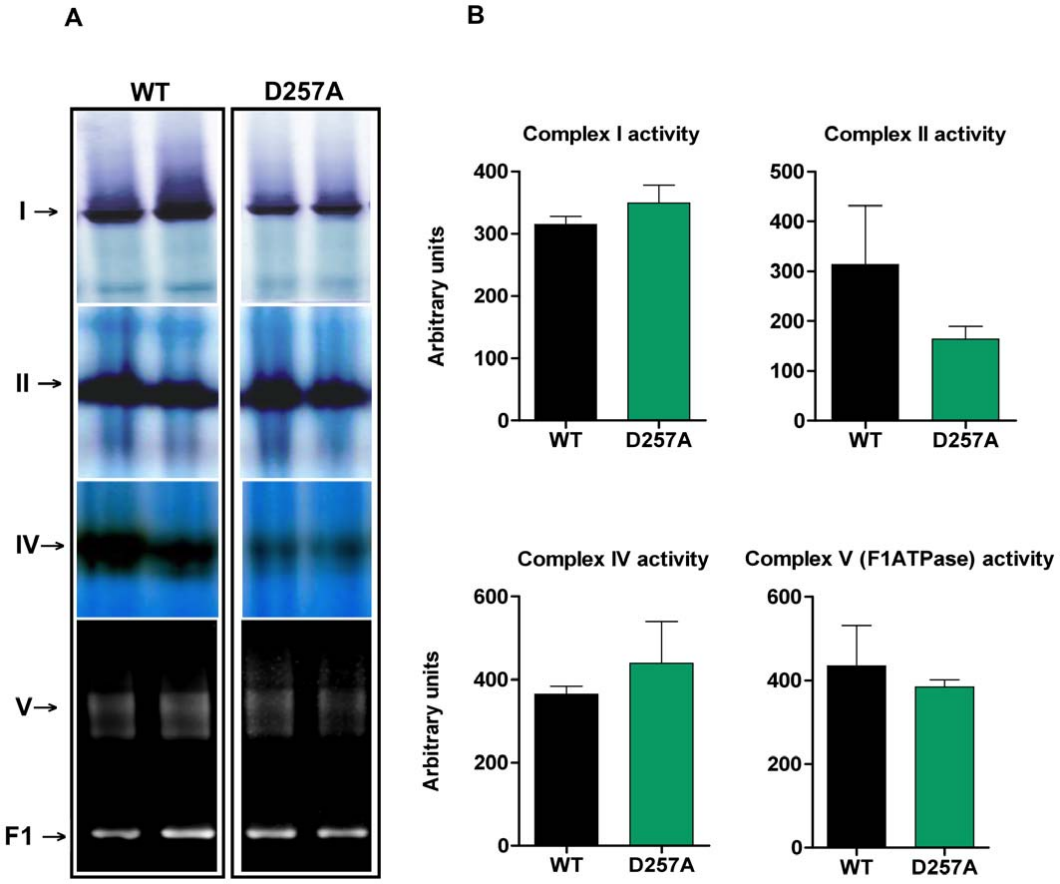
No differences between WT and D257A mice in ETC complexes I, II, IV and F1-ATPase specific activity. (A) The activity of ETC complexes I, II, IV and the F1 domain of the ATPase, in skeletal muscle of 11-mo old WT and D257A mice (n = 7 per group), was determined using Blue Native PAGE, followed by enzymatic colorimetric reactions performed on the gels. Representative blots are depicted. (B) Statistical analysis of ETC complexes specific activity. Arbitrary units represent activity densitometry values normalized to respective content densitometry values for each sample. No statistically significant differences were observed. Mean values ±SEM are shown.

The absolute activities of complexes I and IV (partly mtDNA-encoded) appeared to be greatly reduced in the mutant mice (Fig. 5A) whereas for the all-nuclear-encoded complex II, and ATPase F1 domain, we observed no apparent differences between genotypes (Fig. 5A). When we normalized the activity for each sample to the respective respiratory complex content (specific activity), however, we observed no significant differences between WT and D257A mice for all complexes evaluated (Fig. 5B): Complex I (WT: 314.50±13.56 arbitrary units vs. D257A: 349.10±28.80, p = 0.29), complex II (WT: 313±118.90 vs. D257A: 163.80±26.30, p = 0.26), complex IV (WT: 364.70±19.60 vs. D257A: 440±100.50, p = 0.49), F1 domain of ATPase (WT: 435.10±96.50 vs. D257A: 384.30±18, p = 0.62). We note that large variability in the specific activity in some animal groups (see WT group analysis of complex II activity) may have affected our ability to detect significant changes.

### D257A mice show decreased content of both nuclear-encoded and mitochondrial-encoded ETC subunits and total mitochondrial protein

**Figure 6:**
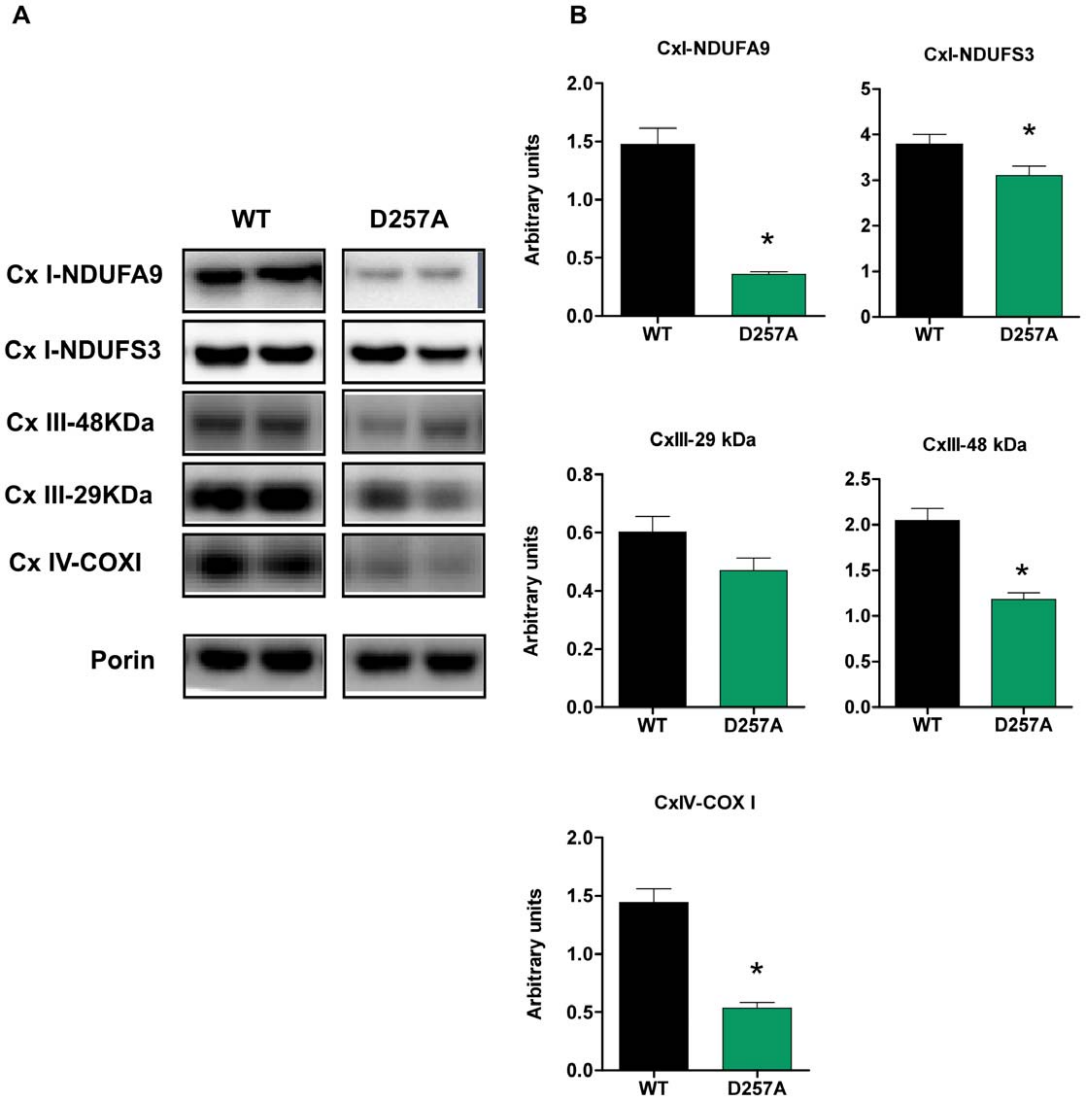
D257A mice show decreased protein expression of both nuclear-encoded and mitochondrial-encoded ETC subunits. (A) The content of selected nuclear-encoded (NDUFA9 and NDUFS3 subunits of Cx I, 29 kDa and 48 kDa subunits of Cx III), and mitochondrial-encoded subunits (COX I subunit of Cx IV) in skeletal muscle extracts of 11-mo old WT and D257A mice (n = 11 per group) were evaluated by Western Blotting. Representative blots are depicted. (B) Statistical analysis of ETC subunits protein expression. Arbitrary units represent densitometry values normalized to porin. Mean values ±SEM are shown. *p<0.05. Cx: complex.

In addition to measuring the content of fully assembled and enzymatically active ETC complexes, we further determined the content of selected individual subunits from each complex by Western blotting experiments. We evaluated the subunits NDUFA9 and NDUFS3 from complex I, both of which are nuclear-encoded (Fig. 6A). We also evaluated two nuclear-encoded subunits from complex III, 29 kDa and 48 kDa (Fig. 6A). Lastly, we evaluated the mitochondrial-encoded COX1 subunit from complex IV, which is a part of the active redox center of this complex, and is essential for catalysis (Fig. 6A). We observed a significant down-regulation of protein expression in the D257A mice compared to WT for all subunits evaluated, whether nuclear- or mitochondrial-encoded (NDUFA9-WT: 1.47±0.14 arbitrary units vs. D257A: 0.36±0.02, p<0.0001), (NDUFS3-WT: 3.80±0.20 vs. D257A: 3.10±0.20, p = 0.03) (Complex III 48 kDa-WT: 2.05±0.13 vs. D257A: 1.20±0.07, p<0.0001) (COX1-WT: 1.44±0.12 vs. D257A: 0.53±0.05, p<0.0001), with the exception of the 29 kDa subunit of complex III, which only tended to decrease (WT: 0.60±0.06 vs. D257A: 0.47±0.04, p = 0.07) (Fig. 6B).

In addition, we compared the mitochondrial protein yield in skeletal muscle of 11-mo and 13-mo old WT mice and D257A mice using a standard Bradford assay. At 11 months of age, we observed a significant reduction (−35%) in mitochondrial protein yield of D257A muscle (WT: 4±0.14 vs. D257A: 2.60±0.06 mg of mitochondrial protein/gram of muscle tissue, p = 0.0044). Additionally, we found that as the mutant mice approach their mean lifespan of ∼14-mo, mitochondrial protein yield is even more drastically reduced; by 13-mo of age we detected a 45% decrease compared to WT (WT: 4.30±0.14 vs. D257A: 2.35±0.20 mg of mitochondrial protein/gram of muscle tissue, p<0.0001), suggesting that mitochondria are progressively eliminated in skeletal muscle of D257A mice.

### Mitochondrial bioenergetics is impaired in D257A mice

**Figure 7:**
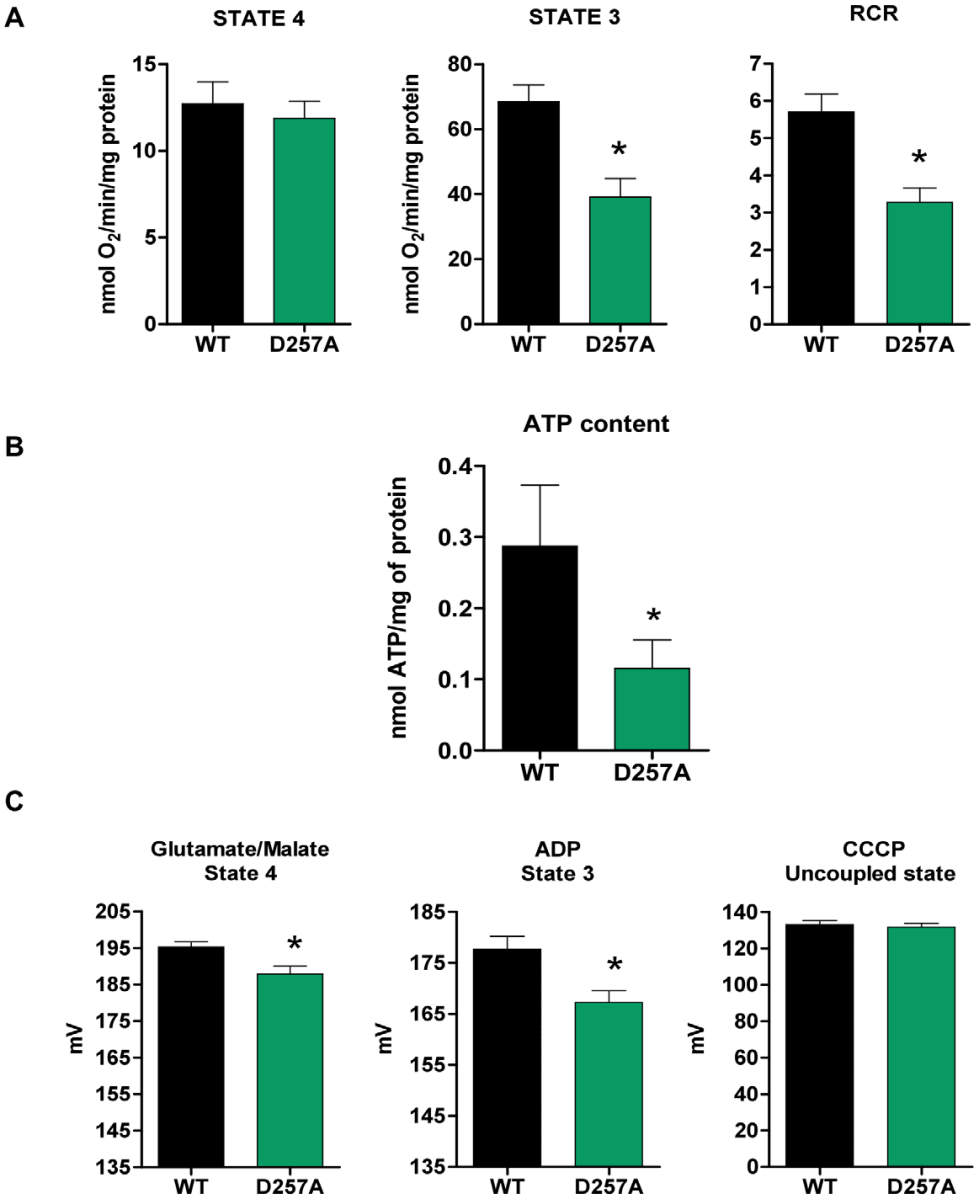
Mitochondrial bioenergetics is compromised in D257A skeletal muscle leading to a drop in mitochondrial Δψ_m_. (A) We determined the effects of mtDNA mutations on O_2_ consumption of skeletal muscle mitochondria obtained from 11-mo old WT and D257A mice (n = 11 per group). Oxygen consumption was measured during state 4 (non-phosphorylative state) with pyruvate/malate as substrate, and during state 3 (phosphorylative state) with the addition of ADP. The respiratory control ratio (RCR), an index of mitochondrial coupling and metabolic activity, was calculated by dividing state 3 by state 4 respiration values. Mean values ±SEM are shown. *p<0.001. (B) We evaluated ATP content in skeletal muscle mitochondria of 11-mo old WT (n = 11) and D257A (n = 8) mice using a luciferin-luciferase based bioluminescence assay. Mean values ±SEM are shown. *p<0.05. (C) Changes in Δψ_m_ were followed qualitatively by monitoring the fluorescence of TMRM that accumulates in energized mitochondria of 13-mo old WT and D257A mice (n = 6 per group). Δψ_m_ was measured during both state 4 (non-phosphorylative state) with glutamate/malate as substrate and during state 3 (phosphorylative state) with the addition of ADP. Measurement of Δψ_m_ after addition of CCCP served as a control for TMRM loading in mitochondria. Mean values ±SEM are shown. *p<0.02.

At 11 months, O_2_ consumption during state 4, the resting state of the mitochondria, did not differ between genotypes (WT: 12.70±1.30 vs. D257A:11.90±0.95 nmol O_2_/min/mg protein, p = 0.31) (Fig. 7A). This was not unexpected because O_2_ consumption during this state is usually minimal. During the phosphorylative state of the mitochondria, state 3 respiration, mutant mitochondria displayed a marked decrease (−43%) in oxygen consumption (WT: 68.40±5.10 vs. D257A: 39±5.80 nmol O_2_/min/mg protein, p = 0.0006) (Fig. 7A), which also led to a significantly lower respiratory control ratio (RCR: −43%) (Fig. 7A) (WT: 5.70±0.49 vs. D257A: 3.27±0.39, p = 0.0005). The RCR is used as an index of mitochondrial coupling and metabolic activity. In the case of D257A mitochondria, the significant decrease in RCR with unaltered state 4 respiration denotes a low metabolic activity due to the defects within the ETC complexes (the reduced content) that lead to overall lower oxidative capacity.

### D257A mice display decreased ATP content

ATP content was significantly lower in D257A mice compared to WT (WT: 0.29±0.08 vs. D257A: 0.11±0.04 nmol ATP /mg protein, p = 0.046) (Fig. 7B). It is apparent that the loss of ETC complexes (Fig. 3A, 3B) can have an impact on ATP content. Therefore, if ETC content is reduced in D257A muscle relative to the amount of total mitochondrial protein, as we have observed, it is logical to expect that the ATP content per amount of total mitochondrial protein would be reduced due to fewer ETC complexes per mitochondrion. Fewer ETCs cause a decrease in state-3 capacity (Fig. 7A), which may provoke a decrease in phosphorylation.

### Mitochondrial membrane potential is significantly lower in D257A mice

We determined the effect of increased mtDNA mutational load on Δψ_m_ of 13-mo old WT and D257A skeletal muscle mitochondria. Δψ_m_ was significantly lower in D257A mice during both state 4 (WT: 195.20±1.40 mV vs. D257A: 187.90±2.15 mV, p = 0.017) (Fig. 7C) and state 3 respiration (WT: 177.70±2.50 vs. D257A: 167.30±2.25 mV, p = 0.01) (Fig. 7C). This drop in Δψ_m_ is a consequence of the decreased proton transport that results from the reduction in functional ETC complexes and could be the trigger for the apoptosis we detected in the mutant mice (see below for discussion of apoptosis).

### Skeletal muscle mitochondria from D257A mice produce significantly fewer ROS

**Figure 8:**
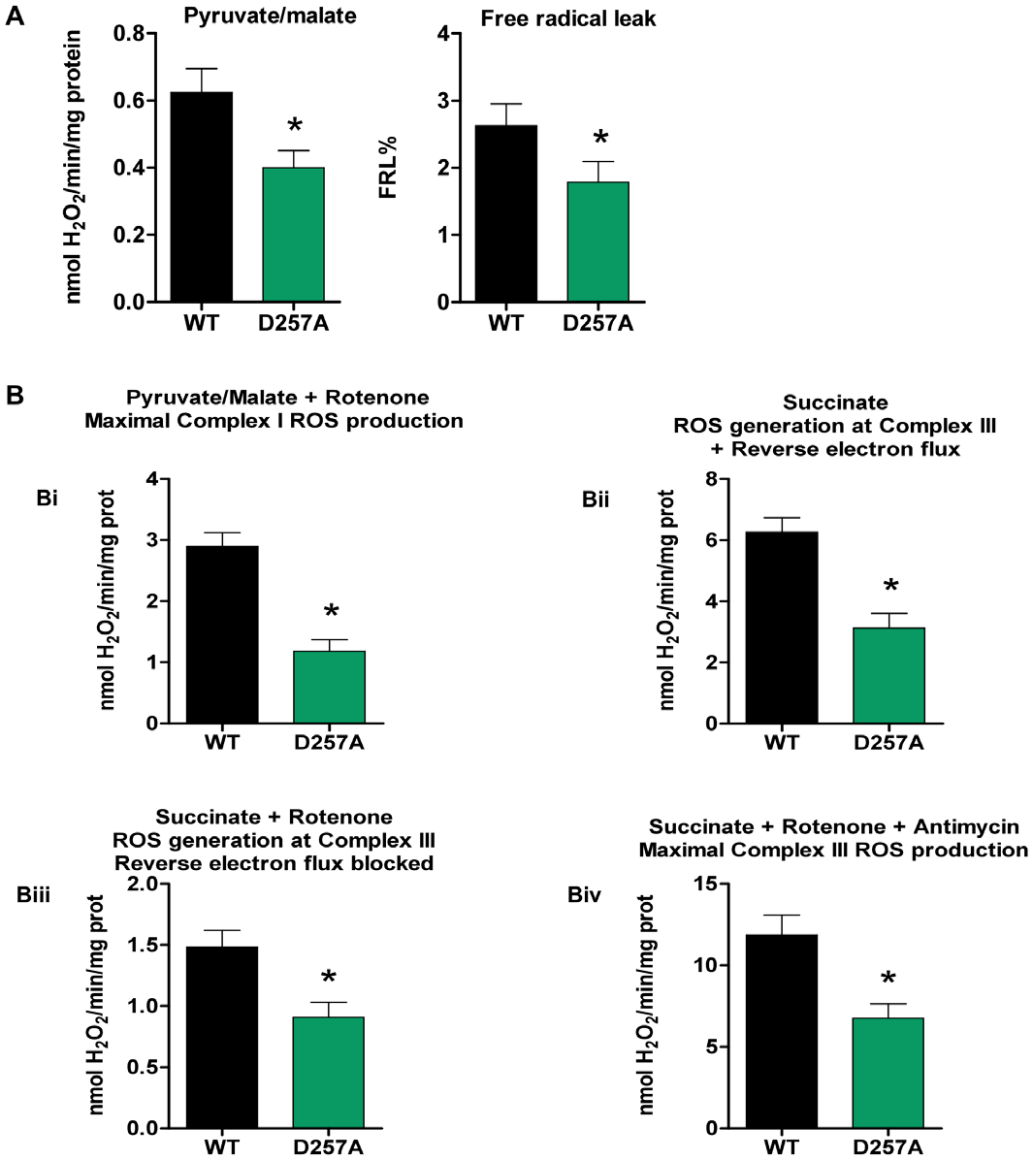
D257A mitochondria produce fewer ROS in both main ROS generators (Complex I and Complex III). (A) We measured H_2_O_2_ production using a sensitive fluorometric assay. Skeletal muscle mitochondria were obtained from 11-mo old, WT and D257A mice (n = 11 per group) and supplemented with pyruvate/malate as substrate for oxidative phosphorylation. Pyruvate/malate was used to study complex I ROS production (under near physiological conditions) which also represents total basal mitochondrial ROS production. Free radical leak percent (FRL%), an index of mitochondrial efficiency, was calculated by dividing the H_2_O_2_ value by twice the state 4 respiration value and the result was multiplied by 100 to give a % final value. Mean values ±SEM are shown. *p<0.05 (B) We used inhibitors of the ETC in order to study maximum rates of H_2_O_2_ production from complexes I and III, since they represent the main sites of ROS generation within the mitochondria (n = 11 per group). For complex I maximum rate (panel B*i*) we used rotenone added to pyruvate/malate-supplemented mitochondria. For complex III maximum rate (panel B*iv*) we used antimycin A plus rotenone, added to succinate- supplemented mitochondria. We also used mitochondria supplemented with succinate alone in order to study complex III ROS production under near physiological conditions (panel B*ii*). In addition, some of the assays with succinate as substrate were performed in the presence of rotenone (panel B*iii*), in order to avoid the backwards flow of electrons to Complex I. Mean values ±SEM are shown. *p≤0.005.

We measured H_2_O_2_ produced by skeletal muscle mitochondria of WT and D257A mice. H_2_O_2_ production was measured during state 4 respiration because ROS production is higher when electron flow is low (ROS production is nearly negligible during state 3 respiration). At 11-mo of age, H_2_O_2_ production was significantly decreased (−33%) in D257A mice (WT: 0.60±0.07 vs. D257A: 0.40±0.05 nmol H_2_O_2_/min/mg protein, p = 0.01) (Fig. 8A). The decreased H_2_O_2_ production by mutant mitochondria also led to the calculation of a significantly lower free radical leak percent (FRL%) (WT: 2.60±0.30% vs. D257A: 1.80±0.30%, p = 0.04) (Fig. 8A), suggesting that in these mitochondria, the fraction of electrons which reduce O_2_ to ROS at upstream components of the ETC instead of reaching cytochrome c oxidase (COX) to reduce O_2_ to water, is much lower compared to WT.

### D257A mitochondria produce fewer ROS in both main ROS generators of the ETC: Complex I and Complex III

We evaluated site-specific ROS generation in 11-mo old mice and found that muscle mitochondria from D257A mice produce fewer ROS at complex III under basal conditions than do WT mitochondria. This is true whether reverse electron flux through complex I is permitted (WT: 6.30±0.47 vs. D257A: 3.13±0.48 nmol H_2_O_2_/min/mg protein, p = 0.0002) (Fig. 8 Bii) or blocked via the complex I inhibitor rotenone (WT: 1.50±0.14 vs. D257A: 0.90±0.12, p = 0.005) (Fig. 8 Biii). Under maximal conditions, D257A mitochondria have also reduced capacity to generate ROS at both complex I (WT: 2.90±0.23 vs. D257A: 1.20±0.19, p<0.0001) (Fig. 8 Bi) and complex III (WT: 11.90±1.20 vs. D257A: 6.75±0.88, p = 0.003) (Fig. 8 Biv) compared to WT. Moreover, the fact that H_2_O_2_ production is decreased by nearly a factor of 4 for both WT and D257A mice when the reverse electron flux is blocked (comparing Y axis values from Fig. 8 Bii and Biii) signifies that this reverse flow is a significant source of ROS produced by the ETC.

### Mitochondrial DNA mutations cause mitochondrial dysfunction in the absence of increased oxidative stress

**Figure 9:**
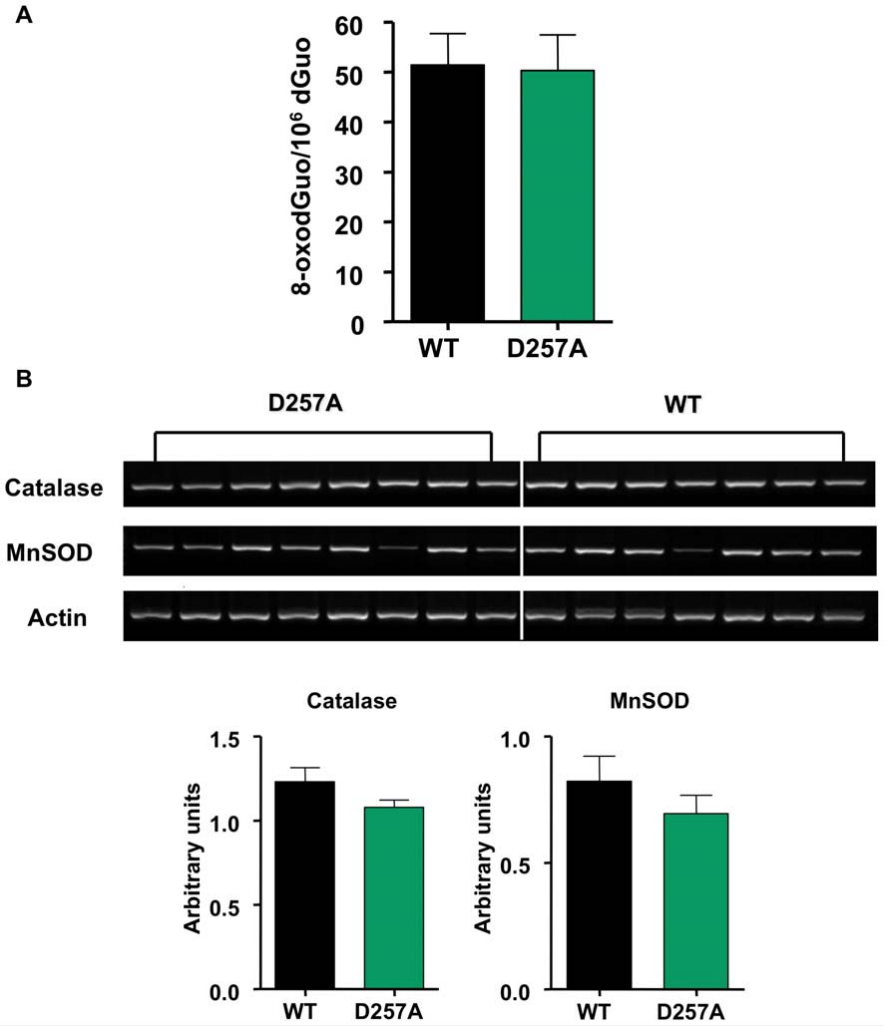
Mitochondrial DNA oxidation levels and antioxidant enzyme mRNA expression remain unaltered in D257A skeletal muscle. (A) We examined a marker of ROS-induced oxidative damage to DNA by assessing the levels of 8-oxodGuo in skeletal muscle mtDNA of 11-mo old WT and D257A mice (n = 11 per group), using HPLC with electrochemical detection. No statistically significant differences were detected. Mean values ±SEM are shown. (B) We measured Catalase and MnSOD mRNA expression in skeletal muscle extracts from 11-mo old WT (n = 7) and D257A (n = 8) mice by RT-PCR. Arbitrary units represent specific mRNA densitometry values normalized to actin mRNA densitometry values. No statistically significant differences were detected. Mean values ±SEM are shown.

In order to correlate our H_2_O_2_ results with further oxidative stress, we next examined a marker of ROS-induced oxidative damage to DNA, by assessing the levels of 8-oxo-7,8-dihydro-2′-deoxyguanosine (8-oxodGuo) in skeletal muscle mtDNA of 11-mo old WT and D257A mice, using HPLC with electrochemical detection. We did not find any differences in the levels of mtDNA oxidation between WT and D257A mice (WT: 51.40±6.30 vs. D257A: 50.30±7.20 8-oxodGuo/10^6^ dGuo, p = 0.9) (Fig. 9A). This is consistent with our previous results, which also showed no significant differences between WT and D257A skeletal muscle in F2-isoprostanes, a marker of lipid peroxidation [20]. Hence, despite increased mutational load, mitochondria from D257A mice do not show increased oxidative stress.

### No difference in antioxidant enzyme mRNA expression between genotypes

We measured mRNA expression of Catalase and the mitochondrial-specific isoform of superoxide dismutase (SOD), MnSOD, via RT-PCR. We found no difference in either Catalase (WT: 1.20±0.08 vs. D257A: 1.10±0.04 arbitrary units, p = 0.1) (Fig. 9B) or MnSOD (WT: 0.80±0.01 vs. D257A: 0.70±0.07 arbitrary units, p = 0.3) (Fig. 9B) between genotypes.

### Apoptosis in D257A skeletal muscle is evident by an increase in cytosolic mono- and oligo-nucleosomes content

**Figure 10:**
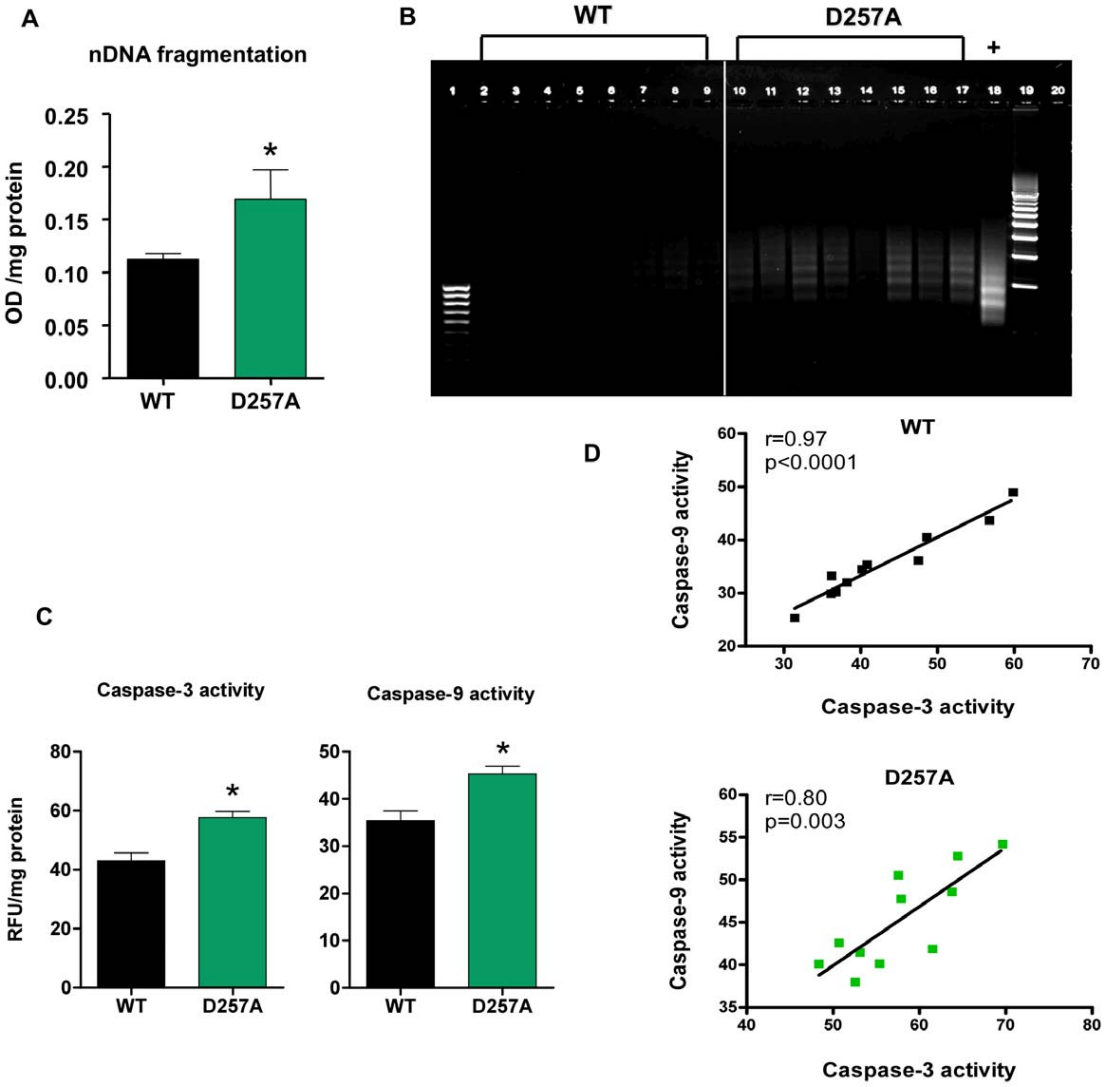
Apoptosis is evident in D257A muscle by increased cytosolic DNA fragments, DNA laddering, and caspase activation. (A) Cytosolic fractions from 11-mo old WT and D257A skeletal muscle (n = 11 per group) were prepared. Nuclear DNA fragmentation was quantified as the amount of mono- and oligo-nucleosomes present in the cytosol, using a sandwich ELISA. Mean values ±SEM are shown. *p<0.05. OD: optical density. (B) DNA from 13-mo old WT and D257A (n = 8 per group) mice was extracted and subjected to a DNA laddering-specific ligation PCR. PCR products were electrophoresed through 1% agarose gels and visualized under UV light for apoptosis-specific DNA ladders of ∼180–200 bp multiples. Lane 1: 100 bp molecular marker. Lanes 2–9: WT PCR products. Lanes 10–17: D257A PCR products. Lane 18: Positive control. Lane 19: 500 bp molecular marker. (C) Cytosolic fractions from 11-mo old WT and D257A skeletal muscle (n = 11 per group) were prepared. Caspase -3 and -9 activities were measured using a fluorometric protease assay kit which is based on detection of cleavage of the substrates DEVD-AFC or LEHD-AFC by caspase-3 and -9 respectively. Mean values ±SEM are shown. *p<0.002. RFU: raw fluorescent units. (D) Caspase-3 activity was correlated with caspase-9 activity in WT and D257A mice (n = 11 per group). Pearson correlation r values are shown in the top right corner. Correlations were significant for both genotypes; WT: r = 0.97, p<0.0001 and D257A: r = 0.8, p<0.003.

We quantified apoptotic DNA fragmentation by measuring the amount of mono- and oligo-nucleosomes released into the cytosol, using a quantitative “Cell Death” detection ELISA (Roche, Germany). This assay detects the nucleosomal DNA digestion products of ∼180–200 bp or multiples thereof that are generated during apoptosis. We observed a significant release of these fragments into the cytosol of D257A muscle (WT: 0.110±0.006 vs. D257A: 0.170±0.030 OD/mg protein, p = 0.035) (Fig. 10A).

### DNA laddering is evident in skeletal muscle of D257A mice

We corroborated the results of the cell death ELISA by employing an alternate method for detecting apoptotic nucleosomal DNA fragments, the DNA laddering assay. Prominent DNA ladders are evident in muscle extracts from the D257A mice while such ladders are not observed in WT mice (Fig. 10B).

### Caspase-3 and caspase-9 activities are significantly higher in D257A mice

We measured caspase-3 activity in the cytosol of 11-mo old WT and D257A muscle and found that it is significantly higher in the mutant mice (WT: 43±2.70 vs. D257A: 57.70±1.97 RFU/mg protein, p = 0.0003) (Fig. 10C). Similarly, caspase-9 activity showed a significant increase in D257A muscle compared to WT muscle (WT: 35.40±2 vs. D257A: 45.30±1.70 RFU/mg protein, p = 0.0014) (Fig. 10C). Activation of caspase-9 was significantly correlated with increased caspase-3 activity for both WT (r = 0.97, p<0.0001) and D257A mice (r = 0.80, p = 0.0029) (Fig. 10D). These results reveal the induction of the mitochondrial-mediated pathway of apoptosis because activation of the mitochondrial pathway-specific caspase-9 leads to further cleavage and activation of caspase-3, which is directly responsible for the downstream events (*i.e.*, cleavage of endo-nucleases and DNA repair enzymes) that lead to apoptosis.

## Discussion

The accumulation of multiple mtDNA point mutations and deletions coincides with the age-dependent loss of muscle fibers in multiple mammalian species [18], [27], [28], [29], [30]. Studies on sarcopenia in rodents and humans, using laser capture microdissection show that mtDNA deletions colocalize with ETC dysfunction in specific fiber regions [14], [31], with fibers manifesting the phenotype when the ratio of deleted mtDNA genomes is above 80% [31]. Interestingly, mtDNA mutations are absent from phenotypically normal regions within individual muscle fibers [32]. Similarly, in aged (69–82 years old) human muscle, clonally expanded base-substitution mutations and deletions in mtDNA are associated with a deficiency in the mitochondrially encoded cytochrome c oxidase (COX) [33]. Although ETC deficiency is only observed in fiber regions that cointain high levels of mtDNA deletions, it has been proposed that extension of the ETS-abnormal region eventually extends throughout the length of the fiber leading to fiber athrophy, breakage and loss [31]. Although these studies do not prove causality, they provide evidence in support of the hypothesis that mtDNA mutations and deletions contribute to the development of sarcopenia. The question of correlation versus causality has recently begun to be addressed by the generation of mitochondrial mutator strains to investigate the role of high mtDNA mutational load on sarcopenia [20]. Here, we extend these observations to characterize in detail the sarcopenic phenotype and mitochondrial respiratory function in skeletal muscle from mutator mice.

Type I and type II muscle fiber numbers were not dramatically different in D257A mice compared to WT control, at least in the gastrocnemius muscle. We did find evidence of muscular atrophy by 13 months in D257A gastrocnemius, represented by an ∼20% decrease in type I fiber diameter in these animals. A more dramatic effect of mtDNA mutations accumulation on type I fibers is consistent with the higher mitochondrial density, and reliance on oxidative phosphorylation in these so-called “slow twitch” fibers relative to classic “fast twitch” type II (particularly type IIb) fibers. Remarkably, the pattern of type I fiber diameter reduction in D257A mice is different than that observed in rodents [34], and humans [35], which are associated with type II fiber atrophy with age. Possibly, the very high load of mtDNA mutations in D257A mice as compared to normally aged WT mice, leads to preferential dysfunction of type I fibers, which rely heavily on oxidative phosphorylation. The difference in fiber type alterations in D257A mice and normal aging could also be interpreted as evidence that mtDNA mutations are not the initiating event in fiber atrophy and sarcopenia in humans. Different mechanisms of muscle aging in D257A and wild-type mice are also supported by the DNA microarray analysis, which is characterized by an extensive induction of p53-induced apoptotic genes in aged wild-type muscle [25], but not in D257A mice as reported here. This may reflect an increased contribution of nuclear genomic instability in normal aging, as compared to aging of D257A mice. However, we also note that the type of mtDNA lesions may play a role in the different observations of D257A mice and normal aging. Importantly, in D257A mice mtDNA point mutations appear to be the predominant lesion [26], whereas mtDNA large deletions are associated with regional fiber dysfunction in humans [31].

We found robust decreases in mitochondrial state 3 (phosphorylative state) O_2_ consumption and significantly lower mitochondrial ATP content in gastrocnemius and quadriceps muscles from D257A mice, compared to WT animals. D257A mitochondria were profoundly less metabolically active than controls, as reflected by respiratory control ratios (state 3/state 4 O_2_ consumption) of less than 3.5. These findings clearly indicate that oxidative phosphorylation is compromised in skeletal muscle of mutant mice and provide a causal role for mtDNA mutations in skeletal muscle mitochondrial dysfunction. It should be noted that decreases in ATP synthesis and state 3 respiration during normal aging are all well-documented for multiple species and tissues, including human skeletal muscle [36], [37], [38], [39], [40], [41]. Our findings in skeletal muscle are also consistent with those of Trifunovic and colleagues, which demonstrated a severe decline in mitochondrial respiration in mouse embryonic fibroblasts and impaired ATP production in the hearts of similar mutator mice [21], [26], [42].

These defects in oxidative phosphorylation are likely the cause of the drop in mitochondrial membrane potential (Δψ_m_) that we have detected in mitochondria from mutant mice. Low Δψ_m_ has also been observed in mitochondria isolated from normally-aged (∼20–30 mo) rodents and from fibroblasts of elderly (≥60 yr) human subjects [43], [44], [45], [46], [47], and was found to correlate with reduced ATP synthesis. A reduction in Δψ_m_ leads to matrix condensation and release of pro-apoptotic factors into the cytosol, facilitating cell death [48]. In our D257A model, the drop in Δψ_m_ was indeed accompanied by up-regulation of apoptosis, as evidenced by an increased release of mono- and oligo-nucleosomal fragments into the cytosol, prominent DNA laddering and up-regulation in the activities of caspase-9 and caspase-3. The observation of increased caspase-9 activity as well as the significant positive correlation between caspase-3 and caspase-9 activity in D257A mice suggests that activation of the mitochondrial pathway is at least partly responsible for the apoptosis we detected in D257A muscle. Accelerated skeletal muscle apoptosis has been well documented with aging [49], [50] and we previously showed that increased caspase-3 cleavage is characteristic of skeletal muscle during normal aging [20]. Furthermore, previous studies have suggested that age-related apoptosis and/or necrosis in response to energy depletion may occur through activation of the mitochondria-mediated signaling pathway [48], [51], [52], [53].

Accumulation of mtDNA mutations and deletions will likely have a direct impact on the transcription and translation of ETC complexes and may preclude the assembly of functional complexes within the inner mitochondrial membrane. Decreases in the activity of ETC complexes with age, and in association with accumulated mtDNA mutations/deletions, are documented in skeletal muscle of rodents and humans [14], [54], [55], [56], [57], [58]. Interestingly, D257A mice showed a significant decrease in the content of fully assembled, enzymatically active complexes I, III and IV, all of which contain subunits encoded by mtDNA, while the content of all nuclear-encoded complexes II and F1 domain of ATPase showed no difference between genotypes. This confirms our hypothesis that mtDNA mutations have a direct impact on the assembly of functional ETC complexes that are comprised of mtDNA-encoded subunits. Importantly, when ETC complex specific activity was determined, we did not detect significant differences between genotypes in any of the complex activities, suggesting that either only functional complexes assemble or dysfunctional complexes are effectively degraded. However, this remains to be determined. Nonetheless, even if the activity of the remaining ETC complexes in mutant mitochondria is normal, the reduced amount of functional complexes still creates energy deficits resulting in mitochondrial dysfunction. A point that requires special attention regarding measurements of ETC complex enzymatic activities is how the complex activities are expressed. In most cases, enzymatic activities are normalized to total protein content [37], or expressed as a ratio to nuclear-encoded citrate synthase activity [57]. In such cases, the overall activity per amount of mitochondrial protein may not reflect decreases in the actual activity of the individual complexes, but instead decreases in the abundance of the complex [37]. We determined specific activity, as well as complex abundance in order to gain a more precise understanding of the specific defects occurring within D257A mitochondria. Thus, our results show that ETC complexes do not assemble as often in D257A mutant mitochondria. However, those complexes that do manage to assemble have normal activity, perhaps because complexes containing altered proteins might be short-lived.

We also observed a significant down-regulation of mitochondria-related gene sets, and also ETC complex subunits expression in the D257A muscle, whether nuclear- or mitochondrial-encoded. The reduced expression of nuclear-encoded subunits may be the consequence of a reactive adaptation of the nucleus. In agreement with this hypothesis, Alemi *et al.* demonstrated that pathogenic mtDNA deletions had a strong negative effect on nuclear-encoded mitochondrially targeted genes [59]. They proposed that the nucleus senses the irreversible depletion of mtDNA-encoded subunits and responds by down-regulating the interacting subunits that would normally form a functional complex. This proposed down-regulation of nuclear-encoded genes related to mitochondria is fully supported by our gene expression findings, and is likely to exacerbate the mitochondrial defect initiated by the mtDNA mutations.

Besides a reduction in the abundance of ETC complexes, we further detected significantly lower mitochondrial protein yield in the mutant mice. Age-related decreases in mitochondrial protein yield in rodent skeletal muscle have been previously reported [60], [61], but this finding is not uniform [62]. By 11-mo of age, we detected a 35% reduction and at ∼13 months, an even more dramatic 45% reduction in mitochondrial protein yield from D257A muscle, suggesting that mitochondrial content is continuously lost in these animals as they are approaching their mean lifespan of ∼14 months. Because we observed decreased ETC complex content and reduced mitochondrial protein yield in D257A muscle with age, we speculate that this may reflect the selective elimination of dysfunctional, mutation-bearing mitochondria containing the fewest functional ETCs.

The hypothesis that aging is due in part to mtDNA damage and associated mutations [12], [13] was based on the observations that mtDNA is located in the organelle that generates the most cellular ROS and it is relatively unprotected from ROS damage compared to nuclear DNA [6], [7]. The main tenet of the free radical theory of aging [63] is that aging is due to the progressive accrual of ROS-inflicted damage, including mtDNA mutations, the accumulation of which has been postulated to lead to a “vicious cycle” of further mitochondrial ROS generation and mitochondrial dysfunction [12], [13]. The increased production of ROS as a consequence of mtDNA mutations has been demonstrated in some circumstances [64], [65]. In contrast to these studies, however, we and others have clearly demonstrated that mitochondrial mutator mice do not have increased levels of oxidative stress [20], [42], [66]. In this study, we further report that in D257A skeletal muscle, mitochondrial ROS production is significantly decreased as compared to WT tissue. Both the basal and maximal ROS production at complex I and at the proton motive Q cycle operating in complex III – the two major ROS sites within the ETC – were significantly lower in mutant skeletal muscle. These results suggest that ROS production is regulated in a tissue-specific way, at least in the D257A model, and does not necessarily play a role in the increased sensitivity to apoptosis. Importantly, we also found no up-regulation in either MnSOD or Catalase mRNA levels, substantiating that ROS levels are decreased in mutant mice due to a lower ROS production and not due to an up-regulation in antioxidant defenses.

In agreement with the ROS data, we found that muscle mtDNA oxidative damage, as measured by 8-oxodGuo, was not different between genotypes. This agrees with our previous 8-oxodGuo data in D257A liver [20] as well as with oxidative stress data reported by others in liver and heart using D257A mutator mice or mice with a heart-specific mitochondrial mutator defect [42], [66]. Note that we did not detect a significant change in 8-oxodGuo levels, despite a drop in H_2_O_2_ production which may implicate non-ETC and extramitochondrial sources of ROS in mtDNA damage [67]. Furthermore, the paired observations of decreased ROS production and a lowered Δψ_m_ in D257A mice are consistent with the widely accepted notion that increased ROS generation occurs at high mitochondrial Δψ_m_s [68], [69].

We do not postulate that chronic ROS production and oxidative stress do not contribute to mtDNA damage and mutagenesis in the context of normal aging. In fact, expression of a mitochondrially targeted catalase in wild-type mice suppresses age-related mtDNA mutations and deletions [70], [71]. Our results from the D257A model, however, clearly support the idea that in skeletal muscle, mtDNA mutagenesis does not lead to further increases in ROS production and oxidative stress and therefore, these are not important mediators of apoptosis in this animal model. Hence, based on our collective results, we propose instead that respiratory chain dysfunction *per se* is the primary inducer of the sarcopenic phenotype.

We note the observations of Bandy and Davison, the first investigators to put forward a mechanistic elaboration of the mitochondrial “vicious cycle” theory. While they showed that some mtDNA mutations may stimulate ROS production, they also carefully noted that mutations preventing the synthesis of cytochrome b would actually abolish any superoxide production at complex III that normal mitochondria might exhibit, because without cytochrome b in place, complex III cannot be assembled [72]. Later studies also reported that cells possessing large mtDNA deletions would indisputably preclude assembly of both the enzyme complexes known to be responsible for mitochondrial ROS production, complexes I and III [27], [73], [74], [75]. Our findings of lower complex I and III content in combination with lower complex I and III ROS production are in agreement with those studies.

We note that there is debate on the contributing role of point mutations as opposed to deletions in the manifestation of the premature aging phenotypes in mitochondrial mutator mice. Interestingly, heterozygous D257A animals are able to sustain a 500-fold higher mtDNA mutation burden than WT mice without any obvious features of rapidly accelerated aging, indicating that mtDNA point mutations do not limit the natural lifespan of WT mice [70]. We have previously shown that mtDNA deletions accumulate at an accelerated rate in tissues of homozygous D257A (the mice used in this study), and postulated that they may represent the main driving force behind the shortened lifespan and the various aging phenotypes in these mice [71]. Moreover, clonally expanded mutations were also evident in D257A mice as demonstrated by cells with a COX negative phenotype in various tissues investigated [71]. However, a recent study reports no major reductions in the levels of mitochondrially encoded transcripts in D257A mice, suggesting that large numbers of point mutations (as opposed to deletions) underlie the phenotypes of D257A mice [26]. In our view, it is likely that both mtDNA deletions and point mutations contribute to the phenotypes observed in homozygous D257A mice, but that these lesions may have different abundance and consequences in different tissues.

The D257A mice are the first *“in vivo”* mammalian system that allows detailed examination of the causal role of mtDNA mutations in skeletal muscle aging. We observed no differences between genotypes in muscle mass [20], caspase-3 cleavage [20], gene expression patterns, mitochondrial bioenergetics (Fig. S1) and ROS production (Fig. S2) at an early age (3-mo), suggesting that the sarcopenic phenotype in D257A mice is age-induced and not due to developmental defects, underscoring the utility of D257A mice as a model of age-related mitochondrial dysfunction. We propose that the reduction in functional complexes I, III and IV formation in D257A mice represents the primary mechanism responsible for impaired mitochondrial bioenergetics (limited oxidation and phosphorylation capacities). Ultimately, lower electron transfer and decreased proton pumping lead to disturbances in Δψ_m_ and mitochondrial-mediated apoptosis. Importantly, this work is also the first to demonstrate specifically in skeletal muscle, that mtDNA mutations lead to mitochondrial dysfunction concurrent with decreases in ROS production, in contrast to the mitochondrial “vicious cycle” theory of aging. This reduction in ROS is possibly due to: (a) the drop in Δψ_m_, and (b) the less frequent formation of the main ROS generators, complexes I and III. Our findings suggest that accumulation of mtDNA mutations and associated respiratory dysfunction may lead to the activation of apoptosis and loss of irreplaceable cells in susceptible tissues, and therefore may be an important contributor to the aging process.

We note that the D257A model does not represent the multiple aspects of normal skeletal muscle aging, but is a model that addresses the role of mtDNA mutations in age-related mitochondrial dysfunction. Although mtDNA deletions were found to accumulate significantly in tissues of WT mice with age, they accumulate at a 7–11 fold higher level in tissues of D257A mice [70], while point mutations can reach ∼2,500-fold higher occurrence compared to WT mice [71]. Moreover, because cells may have hundreds of mitochondria, and each carries multiple copies of mtDNA likely coexisting as heteroplasmic mtDNA sequences, the contribution of mtDNA mutations and deletions to normal aging phenotypes remains a controversial issue.

## Materials and Methods

### Generation of D257A mice

The generation of *Polg* ^D257A^ transgenic mice has been previously reported [20]. Briefly, the D257A mutation results in a critical residue substitution in the conserved exonuclease domain of POLG, impairing its proofreading ability. Germline transmission of the mutation produced *Polg* ^+/D257A^ mice, which were intercrossed to generate homozygous *Polg* ^D257A/D257A^ mice, hereafter denoted D257A.

### Animals

All animal research was conducted in accordance with the regulatory policies of the Institutional Animal Care and Use Committees of the University of Florida (CL approved protocol #D420) and the University of Wisconsin-Madison (TAP approved protocol #M01265). Male and female WT and D257A mice (initially of a mixed 129Sv/ICR/B6 background before being backcrossed for 4 generations into a C57Bl/6J background) were sacrificed at 11 and 13 months of age, timepoints at which the D257A mice exhibit age-related muscle loss; indeed, muscle loss may be evident as early as ∼9 months of age in these mice [20]. Males and females were pooled for statistical analysis based on our previous observation that there are no differences between sexes in the parameters studied in WT mice of the same background [76]. The animals were housed in quarantines in a climate- and light-controlled facility. After one week of acclimation in the facility the animals were sacrificed by rapid cervical dislocation. Four animals a day were sacrificed. For all of our experiments we used and compared two groups: WT versus D257A mice. For some experiments, 3 mo-old WT and D257A mice were also used (refer to Fig. S1 and S2). Five-mo (n = 15) and 30-mo old (n = 10) WT animals of the same background strain were also used for muscle weight comparisons.

Measurements of number and diameter of muscle fiber. Type I and II fibers were discerned with NADH-tetrazolium reductase stain. Detailed protocols for the stain have been described [22]. The number and diameter of each type fiber were measured in the microscopic images of transverse sections of the gastrocnemius muscles. The diameter was calculated by Image J (NIH) and the data sets from >150 type I fibers and >200 type II fibers were analyzed by ANOVA with Bonferroni's multiple comparison test.

### Mitochondrial and cytosolic isolation

Mitochondrial and cytosolic protein fractions were isolated using differential centrifugation as previously described [76]. Immediately after sacrifice, skeletal muscles from 11-mo old WT and D257A mice (both gastrocnemius and quadriceps muscles were pooled together for all of our biochemical analyses) were extracted, rinsed in saline solution, weighted, then finely minced and homogenized in (1∶5 wt/vol) ice-cold isolation buffer containing 0.21 M mannitol, 0.07 M sucrose, 0.005 M Hepes, 0.001 M EDTA, 0.2% fatty acid-free BSA, pH 7.4, using a Potter-Elvehjem glass homogenizer. The homogenate was centrifuged at 1,000 × *g* for 10 min at 4°C. The supernatant was then centrifuged at 14,000 × *g* for 20 min. After the second spin, the supernatant (crude cytosol) was stored at −80°C and the mitochondrial pellet was resuspended in isolation buffer without BSA and was centrifuged again at 14,000 × *g* for 10 min. The final mitochondrial pellet was resuspended in 350 µl of isolation buffer without BSA.

### Total basal and site-specific ROS production

The rate of mitochondrial H_2_O_2_ production was assayed in freshly isolated mitochondria by a highly sensitive fluorometric method according to Barja [77], and adapted to a microplate reader [76]. H_2_O_2_ generation was monitored by measuring the increase in fluorescence (excitation at 312 nm, emission at 420 nm) due to the oxidation of homovanillic acid by H_2_O_2_ in the presence of horseradish peroxidase. The assay was performed in incubation buffer (145 mM KCl, 30 mM Hepes, 5 mM KH_2_PO_4_, 3 mM MgCl_2_, 0.1 mM EGTA, 0.1% BSA, pH 7.4) at 37°C, and the reaction conditions were: 0.25 mg of mitochondrial protein per mL, 6 U/mL of horseradish peroxidase, 0.1 mM homovanillic acid and 50 U/mL of superoxide dismutase (SOD). The reaction was initiated by the addition of 2.5 mM pyruvate/2.5 mM malate as substrate for oxidative phosphorylation. Pyruvate/malate was used to study complex I ROS production which also represents total basal mitochondrial ROS generation. 5 mM succinate without addition of inhibitors was used to study complex III ROS production, under near physiological conditions, as previously described [76], [77]. We also used inhibitors of the ETC in order to study maximum rates of H_2_O_2_ production from complexes I and III, since they represent the main sites of ROS generation within the mitochondria. For the determination of maximal H_2_O_2_ production by complex I, 2 µM rotenone was added to 2.5 mM pyruvate/2.5 mM malate-supplemented mitochondria. For the determination of maximal H_2_O_2_ production by complex III, 2 µM antimycin A plus 2 µM rotenone were added to succinate-supplemented mitochondria. In addition, some of the assays in which succinate was used as the substrate were performed in the presence of 2 µM rotenone alone, in order to avoid the “backward” flow of electrons to Complex I. After 15 min of incubation at 37°C, the reaction was stopped and the samples were transferred on ice and a stop solution (0.1 M glycine, 25 mM EDTA, pH 12) was added. Known amounts of H_2_O_2_ generated in parallel by glucose oxidase, with glucose as substrate, were used as standards. All samples were run in duplicate.

### Mitochondrial respiration

Mitochondrial oxygen consumption was measured at 37°C by polarography, with a Clark-type oxygen electrode (Oxytherm, Hansatech, Norfolk, UK) under the same conditions used (same mitochondrial preparation, buffer composition and substrate concentrations) for H_2_O_2_ production measurements. The assay was performed in the absence (State 4-resting state) and in the presence (State 3-phosphorylating state) of 500 µM ADP.

### ATP content

Mitochondria isolated from skeletal muscle were used immediately after isolation to determine mitochondrial ATP content with a luciferin-luciferase based bioluminescence assay, following the method of Drew [78]. ATP content methodology was modified from a method of Molecular Probes (A-22066, Eugene, OR, USA). The chemicals used were *D*-luciferin, luciferase (40 µL of a 5 mg/mL solution in 25 mM Tris-acetase, pH 7.8, 0.2 M ammonium sulfate, 15% (v/v) glycerol and 30% (v/v) ethylene glycol), dithiothreitol (DTT), adenosine 5′-triphosphate (ATP), and a Reaction Buffer (10 mL of 500 mM Tricine buffer, pH 7.8, 100 mM MgSO_4_, 2 mM EDTA and 2 mM sodium azide). The reagents and reaction mixture were combined according to the protocol by Molecular Probes. In order to determine ATP content, freshly isolated mitochondria were added to a cuvette containing reaction buffer, *D*-luciferin, luciferase and DTT. In addition, 2.5 mM pyruvate and 2.5 mM malate were added to the reaction mixture, as substrates for oxidative phosphorylation. Known concentrations of ATP standards were used to establish a standard curve. The values for ATP content were normalized to total mitochondrial protein concentration.

### Mitochondrial membrane potential

Δψ_m_ changes in isolated skeletal muscle mitochondria were followed qualitatively by monitoring the fluorescence of tetramethyl rhodamine methyl ester (TMRM, Molecular Probes, Eugene, OR, USA), a cationic lipid-soluble probe that accumulates in energized mitochondria. The method of Scaduto [79] was followed without modification. Briefly, mitochondria (0.25 mg/ml) were incubated at 37°C in a medium composed of 135 mM KCl, 20 mM MOPS, 5 mM K_2_HPO_4_, and 5 mM MgCl_2_ at pH 7.00. The experiment was initiated by the addition of mitochondria to the medium, also containing 0.33 mM TMRM and 5 mM glutamate +2.5 mM malate in order to record Δψ_m_ during state 4. Fluorescence at 546 and 573 nm excitation was monitored using an emission wavelength of 590 nm. This was followed by the addition of ADP (0.17 mM) to record Δψ_m_ during state 3. Addition of 0.5 mM CCCP served as a positive control, to assess Δψ_m_ in completely depolarized mitochondria.

### Calculation of the mitochondrial free radical leak

H_2_O_2_ production and O_2_ consumption were measured in parallel in the same muscle mitochondria under similar experimental conditions. This allowed the calculation of the fraction of electrons which reduce O_2_ to ROS at upstream components of the respiratory chain (the percent free radical leak or FRL%) instead of reaching cytochrome oxidase to reduce O_2_ to water. Because two electrons are needed to reduce 1 mole of O_2_ to H_2_O_2_ whereas four electrons are transferred in the reduction of 1 mole of O_2_ to water, the percent free radical leak can be calculated as the rate of H_2_O_2_ production divided by two times the rate of O_2_ consumption, and the result is multiplied by 100.

### Calculation of the mitochondrial respiratory control ratio (RCR)

Measurement of oxygen consumption using a Clark-type electrode in the absence (State 4) and presence (State 3) of saturating amounts of ADP (500 µM) allowed calculation of the respiratory control ratio (RCR) (State 3/State 4 oxygen consumption) as an indicator of the degree of coupling and metabolic activity of the mitochondrial preparations.

### Determination of mitochondrial protein yield

In order to determine mitochondrial yield, we first determined the total protein concentration in each mitochondrial extract by the Bradford assay. Each concentration value was multiplied by the total volume of each mitochondrial suspension. This product was divided by the skeletal muscle weight used each time to obtain the respective mitochondrial extract. Mitochondrial yield was expressed as total mitochondrial content per gram of skeletal muscle tissue.

### Microarray Analysis

Details regarding sample preparation and array hybridization are described elsewhere [80] (see also Supporting File S1). Briefly, total RNA was isolated from gastrocnemius muscle using TRIZOL (Invitrogen, Carlsbad, CA, USA) and 5 individual samples from each group were used for the DNA microarray experiments. DNA chips were hybridized with synthesized biotin-labeled cRNA and scanned with a GeneArray Scanner (Affymetrix, Santa Clara, CA, USA). We performed Parametric Analysis of Gene Set Enrichment (PAGE) [23] to identify classes of genes that were differentially expressed as a result of mtDNA mutations. Genes were annotated with functional data from the Gene Ontology (GO) consortium (http://www.geneontology.org). We considered GO terms that were annotated at Level 3 or greater and were represented by at least 10 but not more than 1000 genes. A GO term was considered to be significantly changed by treatment if the p-value for both the PAGE and a False Discovery Rate (FDR) analysis was <0.01. We also calculated *Z* ratios for each gene set, which serve as a normalization factor [81].

### Quantitative RT-PCR

Detailed protocols for quantitative RT-PCR analysis have been described [82]. The gastrocnemius muscles were dissected, frozen in liquid N2, and stored at −80°C. Total RNA was extracted from the frozen tissues using the TRIZOL reagent. Detection of mRNA was carried out with the TaqMan EZ RT-PCR kit using an Applied Biosystems 7500 Real-Time PCR System (Applied Biosystems, Foster City, CA, USA). *β*-Actin was used as an internal standard. Oligonucleotide primers and MGB fluorescent probes (TaqMan Gene Expression Assays) were purchased from Applied Biosystems. Samples were run in duplicate.

### Oxidative Damage to mtDNA

Mitochondrial DNA oxidation was measured according to Sanz *et al.* [83], with modification. Briefly, mitochondrial DNA, free of nDNA, were isolated by the method of Latorre *et al.* [84], adapted to mammals [85]. After isolation mtDNA was completely dissolved in 85 µL water containing 30 µM deferoxamine mesylate (Sigma Aldrich, St. Louis, MO, USA), DNA was digested with 4 U of Nuclease P1 (dissolved in 300 mM sodium acetate, 0.2 mM ZnCl_2_, pH 5.3), and 5 U of alkaline phosphatase during 60 min at 50°C. After filtering, samples were put into an autosampler vial at 4°C for HPLC-EC-UV analysis. 8-oxo-7,8-dihydro-2′-deoxyguanosine (8-oxodGuo) and 2′-deoxyguanosine (dGuo) were measured by HPLC with online electrochemical and ultraviolet detection respectively. For analysis, the nucleoside mixture was injected into two Delta-Pak (150×3.9 mm id, 5 µm) C-18 reversed-phase columns (Waters, Milford, MA, USA). 8-oxodGuo was detected with an electrochemical detector (Coulochem III, ESA Inc, Chelmsford, MA, USA) with a PEEK filter protected 5011A analytical cell (ESA, 5 nA; screen electrode: +205 mV; analytical electrode: +275), and dG was measured with a Spectra SYSTEM UV1000 detector (Thermo Electron Corp., San Jose, CA, USA) set at 290 nm. Chromatograms were recorded using EZChrome Elite (Scientific Software INC., Pleasanton, CA, USA). Calibration curves for dGuo and 8-oxodGuo were constructed by injection of each standard 3–4 times. The HPLC buffer consisted of 9% v/v methanol and 50 mM sodium acetate, set to pH 5.3, with acetic acid, then filtered through a CN 0.2 µm filter from Nalgene Nunc (Rochester, NY, USA).

### Blue native polyacrylamide gel electrophoresis (BN-PAGE) analysis for determination of content and enzymatic activity of respiratory complexes

For determination of the content of the ETC complexes, we followed the protocol as described by Schagger *et al*. with some modification [86]. Skeletal muscle was homogenized in buffer 1 containing 20 mM MOPS, 440 mM sucrose, 1 mM EDTA and 0.5 mM PMSF, pH 7.2 at 4°C. The homogenates were centrifuged at 20,000 x *g* for 20 min. The pellet was resuspended in 80 µl of buffer containing 1 M aminocaproic acid, 50 mM Bis-tris and 0.5 mM PMSF, pH 7.0. The membranes were then solubilized by the addition of 30 µl n-dodecylmaltoside (10%, prepared fresh), followed by ultracentrifugation for 25 min at 100,000 x *g*. The supernatant, containing all the solubilized mitochondrial membrane proteins was used for the BN-PAGE. 7 µl of 5% w/v coomassie brilliant blue G-250 in aminocaproic acid (1 M) were added to 100 µl of supernatant and samples were loaded to a gel. For electrophoresis, a 3–12% gradient gel with 4% of stacker was used. The anode buffer was comprised of 50 mM Bis-Tris, pH 7.0. The cathode buffer was comprised of 50 mM tricine, 15 mM Bis tris, and coomassie brilliant blue G-250 (0.02% w/v), pH 7.0. Samples were electrophoresed at 90 V for 20 min, and thereafter at 170 V for 2 h, at 4°C. Immediately after electrophoresis gels were incubated in coomassie brilliant blue G-250 solution (0.1% coomassie in 10% acetic acid and 40% methanol) for 1 h, followed by incubation in de-staining solution (10% acetic acid, 40% methanol) for 2 h. After de-staining, gels were photographed and analyzed using the Alpha Innotech FluorChem SP imaging system (Alpha Innotech, Santa Clara, CA, USA). Densitometry values were normalized to total protein loaded per well, measured by the Bradford assay [87]. For determination of enzymatic activity, enzymatic colorimetric reactions were performed on the BN-PAGE. Gels were incubated overnight at room temperature with the following solutions: Complex I: 2 mM Tris–HCl, pH 7.4, 0.1 mg/ml NADH, and 2.5 mg/ml NTB (nitrotetrazolium blue). Complex II: 4.5 mM EDTA, 10 mM KCN, 0.2 mM phenazine methasulfate, 84 mM succinic acid and 50 mM NTB in 1.5 mM phosphate buffer, pH 7.4. Complex IV: 5 mg 3∶30-Diamidobenzidine tetrahydrochloride (DAB) dissolved in 9 ml phosphate buffer (0.05 M, pH 7.4), 1 ml catalase (20 µg/ml), 10 mg cytochrome c, and 750 mg sucrose. Complex V: 35 mM Tris, 270 mM glycine, 14 mM MgSO_4_, 0.2% Pb(NO_3_)_2_, and 8 mM ATP, pH 7.8. Gels were then washed in distilled water and photographed immediately. Densitometry values for activity were normalized to respective content densitometry values.

### Western blots of selected mitochondrial- and nuclear-encoded subunits from ETC complexes I, III, and IV

The protein content of skeletal muscle mitochondrial respiratory chain complexes was estimated using Western blot analysis. Immunodetection was performed using specific antibodies for the 39 kDa (NDUFA9) and 30 kDa (NDUFS3) subunit of complex I (1∶1000 and 1∶1000, respectively), 48 kDa (CORE 2) and 29 kDa (Rieske iron-sulfur protein) subunits of complex III (1∶1000 and 1∶1000, respectively), and COXI subunit of complex IV (1∶1000) (ref. A21344, A21343, A11143, A21346 and A6403, respectively; Molecular Probes, Eugene, OR, USA). An antibody to porin (1∶5000, A31855, Molecular Probes, Eugene, OR, USA) or beta-actin (1∶5000, AB20272, Abcam Cambridge, MA, USA), as a loading control for total mitochondrial mass or total protein content, was also used. Appropriate peroxidase-coupled secondary antibodies and chemiluminescence HRP substrate (Millipore, MA, USA) were used for primary antibody detection. Signal quantification and recording was performed with a ChemiDoc equipment (Bio-Rad Laboratories, Inc., Barcelona, Spain). Protein concentration was determined by the Bradford method [87]. Data were expressed as Arbitrary Units.

### Determination of MnSOD and Catalase mRNA expression by RT-PCR

In order to extract RNA, skeletal muscles (1/10 weight/reagent volume) were homogenized in 1 mL of Tri reagent (Sigma-Aldrich, St. Louis, MO, USA) and RNA was isolated according to the manufacturer's protocol. One microgram of isolated RNA was reverse transcribed (Eppendorf RT plus PCR kit, Hamburg, Germany) using oligo-(dT) primer, as described by the manufacturer's instructions. PCR was performed on 3-µl aliquots from each cDNA reaction, using primer sets for detecting MnSOD (5′-GGTGGCCTTGAGCGGGGACTTG-3′, 5′-GGTGGGTGGGGAGGTAGGGAGGAT-3′, sense and antisense, respectively) and Catalase (5′-ATGGCCTCCGAGATCTTTTCAATG-3′, 5′-GAGCGCGGTAGGGACAGTTCAC-3′, sense and antisense, respectively). The sizes of the amplification products were 611 bp for MnSOD and 366 bp for Catalase. Conditions for PCR reactions were for MnSOD: 94°C for 30 sec, 58°C for 30 sec, and 72°C for 30 sec and for Catalase: 94°C for 30 sec, 57.7°C for 30 sec, and 72°C for 30 sec. PCR amplification was conducted for 29 cycles for both MnSOD and Catalase. RT-PCR products were analyzed by agarose gel electrophoresis and digital imaging of the ethidium bromide-stained gel, using the Alpha Innotech FluorChem SP imaging system (Alpha Innotech, Santa Clara, CA, USA).

### Enzymatic measurement of caspase-3 and caspase-9 activities

Caspase-3 and caspase-9 activities were measured using fluorometric protease assay kits: (Caspase-3/CPP32, Caspase-9/Mch6, Biovision, Mountain View, CA, USA) according to manufacturer's instructions. Briefly, the assays are based on detection of cleavage of the substrate DEVD-AFC (AFC: 7-amino-4-trifluoromethyl coumarin) by caspase-3, and LEHD-AFC (AFC: 7-amino-4-trifluoromethyl Coumarin) by caspase-9. DEVD-AFC and LEHD-AFC emit blue light (λ_max_ = 400 nm); upon cleavage of the respective substrate by caspase-3 or caspase-9, free AFC emits a yellow-green fluorescence (λ_max_ = 505 nm), which can be quantified using a fluorescence microplate reader. Results were expressed as raw fluorescence units per mg of cytosolic protein.

### Determination of cytosolic mono- and oligonucleosomes

Apoptotic DNA fragmentation was quantified in skeletal muscle by measuring the amount of cytosolic mono- and oligonucleosomes using a Cell Death detection ELISA (Roche, Germany). Briefly, wells were coated with a monoclonal anti-histone antibody. Nucleosomes in the sample bound to the antibody followed by the addition of anti-DNA-peroxidase, which reacted with the DNA associated with the histones. The amount of peroxidase retained in the immunocomplex was determined spectrophotometrically with ABTS (2.2′-azino-di-[3-ethylbenzthiazoline sulfonate]) as a substrate. Results were expressed as arbitrary OD units normalized to mg of cytosolic protein.

### DNA laddering

To enable detection of nucleosomal ladders in apoptotic cells, the DNA ladder assay was performed. Skeletal muscle was homogenized in 1 mL DNAzol (Molecular Research Center Inc., Cincinnati, OH, USA). Proteinase K (Qiagen, Valencia, CA, USA) was added to the homogenates, which, after a 3 h incubation period, were centrifuged (10,000 × *g* for 10 min at 4°C) and the supernatants were precipitated and washed with 100% and 75% ethanol, respectively. After digestion with RNase A, DNA samples were subjected to a DNA ladder-specific ligation-mediated PCR, following the manufacturer's protocol (Maxim Biotech, San Francisco, CA, USA). Briefly, isolated DNA is subjected to an overnight ligation reaction using de-phosphorylated adaptors (12-mer: 5′-AGTCGACACGTG-3′, 27-mer: 5′-GACGTCGACGTCGTACACGTGTCGACT-3′) that are ligated to the ends of DNA fragments generated during apoptosis, using T4 DNA ligase. When the mixture is heated to 55°C, the 12-mer is released. Next, the 5′ protruding ends of the molecules are filled by Taq polymerase. The 27-mer then serves as a primer for PCR in which the fragments with adaptors on both ends are amplified. Conditions for PCR reactions were 72°C for 10 min, 94°C for 1 min, followed by 30 cycles of 94°C for 1 min and 70°C for 2 min. PCR products were electrophoresed through 1% agarose gels containing 0.5 µg/mL ethidium bromide at 80 V for 1 h, and were examined under UV light for the presence of apoptosis-specific nucleosomal ladders.

### Statistical Analyses

All results are expressed as means ± SEM and the means were compared by independent t-tests. Statistical analyses were carried out using the Graph-Pad Prism 4.0 statistical analysis program (San Diego, CA). Statistical significance was set to p<0.05.

## Supporting Information

Table S1 Gene expression changes in gastrocnemius muscle of 13-month-old D257A mice compared to wild type mice.
(0.03 MB XLS)

Figure S1 No differences between WT and D257A skeletal muscle in mitochondrial respiration at 3-mo of age. We determined the effects of mtDNA mutations on O2 consumption of skeletal muscle mitochondria obtained from 3-mo old (n = 8 per group) WT and D257A mice. The respiratory control ratio (RCR), an index of mitochondrial coupling and metabolic efficiency of mitochondrial preparations, was calculated by dividing state 3 by state 4 respiration values. No statistically significant differences were detected. Error bars represent SEM.
(0.15 MB TIF)

Figure S2 No differences in total basal and site-specific reactive oxygen species production at 3-mo of age. Skeletal muscle mitochondria were obtained from 3-mo old (n = 8 per group) WT and D257A mice. We evaluated H2O2 production at complex I in mitochondria supplemented with pyruvate/malate (panel A) because it represents total basal mitochondrial ROS generation. We also used inhibitors of the ETC in order to study maximum rates of H2O2 production from complexes I and III because they represent the main sites of ROS generation within the mitochondria. For measuring the maximal rate of ROS production at complex I (panel B), we added rotenone to pyruvate/malate-supplemented mitochondria. For measurements of the maximum rate of ROS generation at complex III (panel E), we added antimycin A plus rotenone to succinate-supplemented mitochondria. We also used mitochondria supplemented with succinate alone (i.e., in the absence of any respiratory inhibitors) in order to study complex III ROS production under near physiological conditions (panel C). In addition, some of the assays with succinate as substrate were performed in the presence of rotenone (panel D), in order to avoid the backwards flow of electrons through Complex I. Free radical leak (FRL%), an index of mitochondrial efficiency (panel F), was calculated by dividing the H2O2 production value by twice the state 4 respiration value and the result was multiplied by 100 to give a final percentage value. No statistically significant differences were detected. Error bars represent SEM.
(0.44 MB TIF)

Supporting File S1 Minimum information about a microarray experiment check list. Supplemental description of gene expression analysis of skeletal muscle from wild type and mitochondrial mutator mice.
(0.03 MB DOC)
