# Supplementary figures and images for: Mitochondrial DNA Mutations Induce Mitochondrial Dysfunction, Apoptosis and Sarcopenia in Skeletal Muscle of Mitochondrial DNA Mutator Mice
Source: PLoS One. 2010 Jul 7;5(7):e11468. doi: 10.1371/journal.pone.0011468 (PMC2898813; doi:10.1371/journal.pone.0011468)

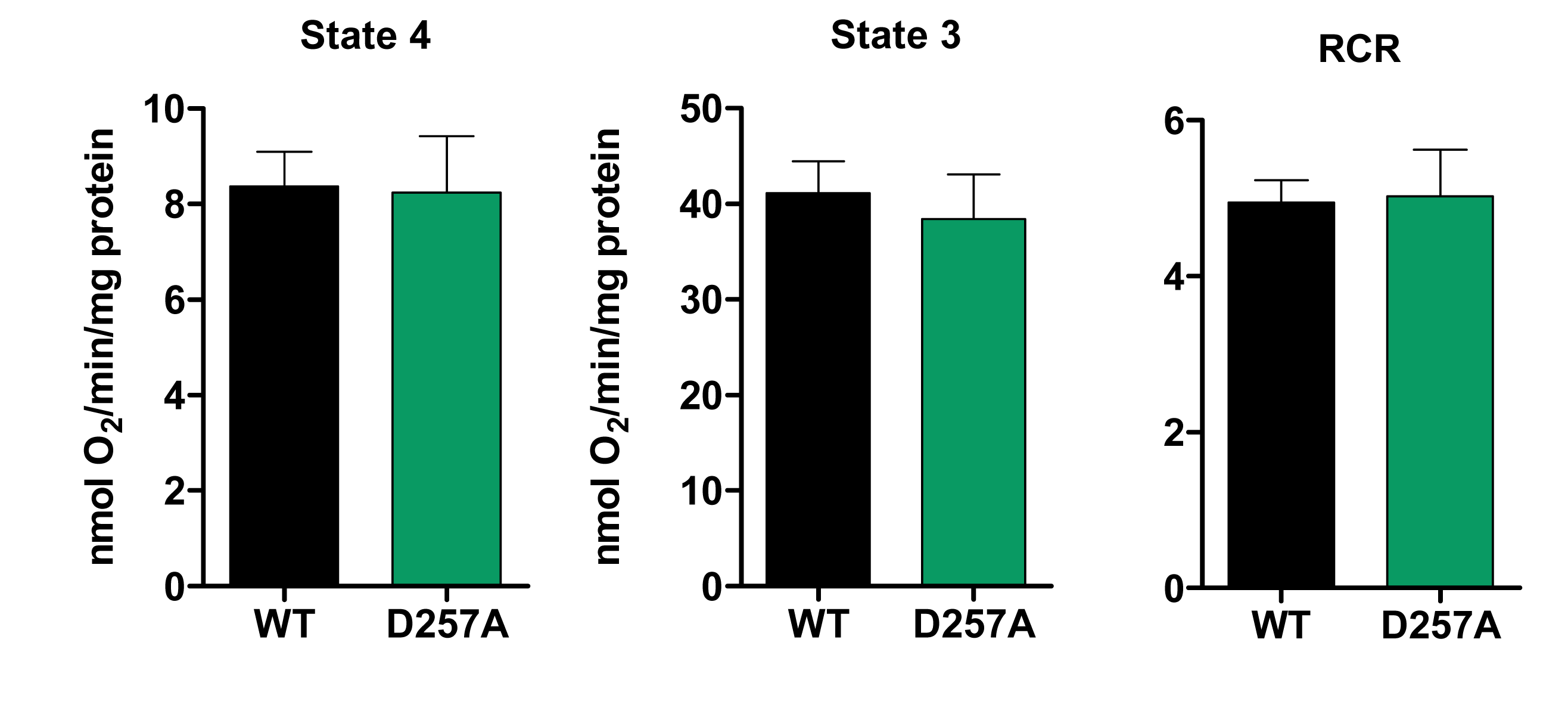

Supplement: Figure S1 — No differences between WT and D257A skeletal muscle in mitochondrial respiration at 3-mo of age. We determined the effects of mtDNA mutations on O2 consumption of skeletal muscle mitochondria obtained from 3-mo old (n = 8 per group) WT and D257A mice. The respiratory control ratio (RCR), an index of mitochondrial coupling and metabolic efficiency of mitochondrial preparations, was calculated by dividing state 3 by state 4 respiration values. No statistically significant differences were detected. Error bars represent SEM. (0.15 MB TIF) [file pone.0011468.s002.tif]

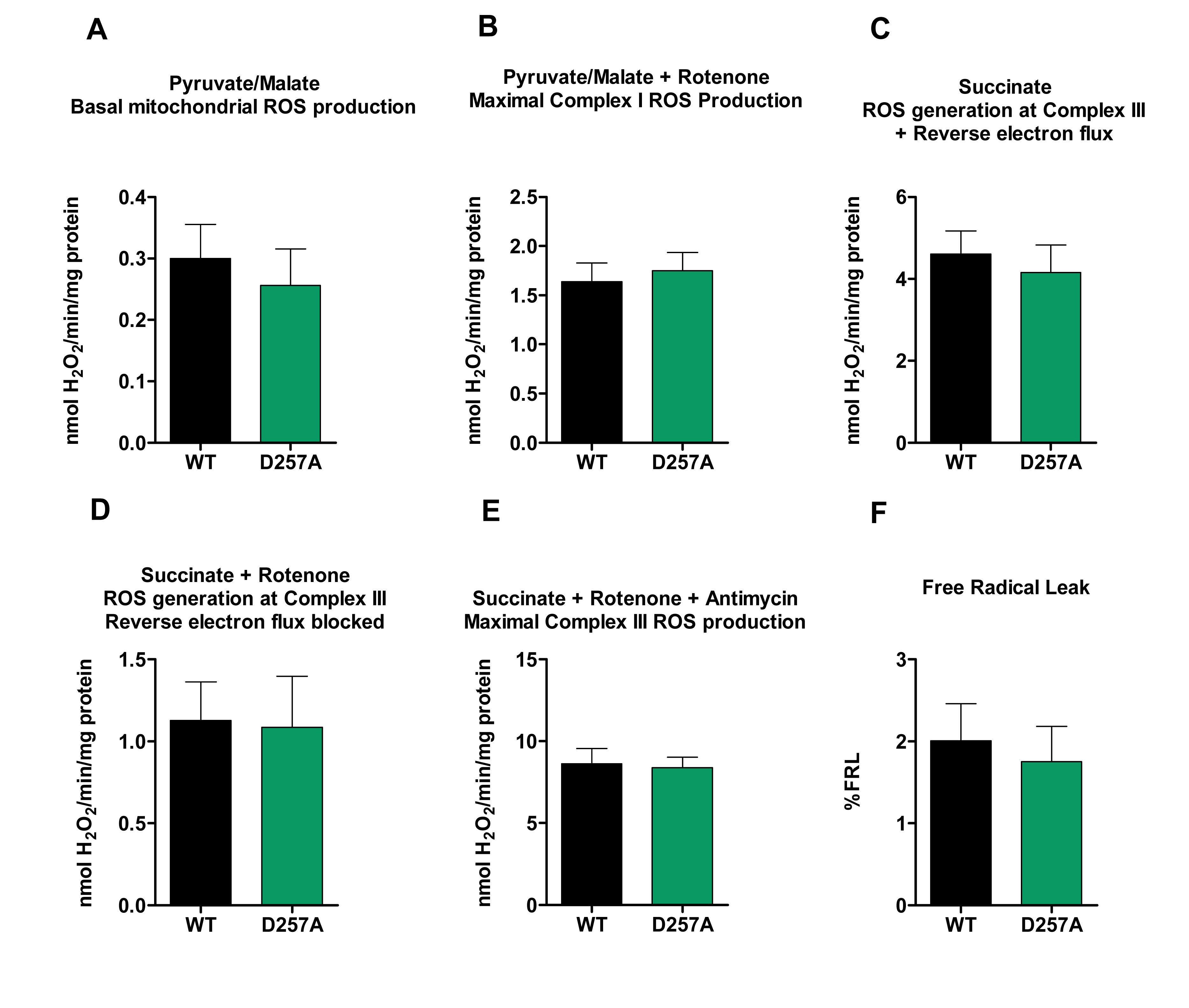

Supplement: Figure S2 — No differences in total basal and site-specific reactive oxygen species production at 3-mo of age. Skeletal muscle mitochondria were obtained from 3-mo old (n = 8 per group) WT and D257A mice. We evaluated H2O2 production at complex I in mitochondria supplemented with pyruvate/malate (panel A) because it represents total basal mitochondrial ROS generation. We also used inhibitors of the ETC in order to study maximum rates of H2O2 production from complexes I and III because they represent the main sites of ROS generation within the mitochondria. For measuring the maximal rate of ROS production at complex I (panel B), we added rotenone to pyruvate/malate-supplemented mitochondria. For measurements of the maximum rate of ROS generation at complex III (panel E), we added antimycin A plus rotenone to succinate-supplemented mitochondria. We also used mitochondria supplemented with succinate alone (i.e., in the absence of any respiratory inhibitors) in order to study complex III ROS production under near physiological conditions (panel C). In addition, some of the assays with succinate as substrate were performed in the presence of rotenone (panel D), in order to avoid the backwards flow of electrons through Complex I. Free radical leak (FRL%), an index of mitochondrial efficiency (panel F), was calculated by dividing the H2O2 production value by twice the state 4 respiration value and the result was multiplied by 100 to give a final percentage value. No statistically significant differences were detected. Error bars represent SEM. (0.44 MB TIF) [file pone.0011468.s003.tif]
