# Supplementary material for: Mitochondrial DNA Mutations Induce Mitochondrial Dysfunction, Apoptosis and Sarcopenia in Skeletal Muscle of Mitochondrial DNA Mutator Mice
Source: PLoS One. 2010 Jul 7;5(7):e11468. doi: 10.1371/journal.pone.0011468 (PMC2898813; doi:10.1371/journal.pone.0011468)
Supplement: Supporting File S1 — Minimum information about a microarray experiment check list. Supplemental description of gene expression analysis of skeletal muscle from wild type and mitochondrial mutator mice. (0.03 MB DOC) [file pone.0011468.s004.doc]

**Supplementary File S1:**

**Minimum Information About a Microarray Experiment**

“Mitochondrial DNA Mutations Induce Mitochondrial Dysfunction, Apoptosis and Sarcopenia in Skeletal Muscle of Mitochondrial DNA Mutator Mice”

Hiona A, Sanz A, Kujoth GC, Pamplona R, Seo AY, Hofer T, Someya S, Miyakawa T, Nakayama C, Samhan-Arias AK, Servais S, Barger, JL, Portero-Otin M, Tanokura M, Prolla TA, Leeuwenburgh CL

*Array design description:*

1) Array used: Affymetrix Mouse Genome 430 2.0 (information regarding the details of this array can be found at www.affymetrix.com)

2) Normalization: All arrays were scaled to 500 to correct for differences in overall signal before analysis in GeneChip® Operating Software (GCOS) 1.3 (Affymetrix). Further analysis and comparisons between experiments was also done in GCOS 1.3 (Affymetrix) and SAM (Significance Analysis of Microarrays) Software.

*Experimental design:*

1) Contact information: Takuya Miyakawa

Departments of Applied Biological Chemistry

University of Tokyo

1-1-1 Yayoi, Bunkyo-ku

Tokyo, 113-8657, Japan

TEL: 81-3-5841-5165

Email: atmiya@mail.ecc.u-tokyo.ac.jp

2) Types of experiments:

a) Effect of mitochondrial DNA (mtDNA) mutations

b) Effect of aging

c) WT (13 month-old *Polg*^+/+^ mice) vs. D257A (13 month-old *Polg*^D257A/D257A^) mice

d) Time course

3) Experimental factors:

a) Type of gene: *Polg*^D257A/D257A^

b) Time (age)

4) Number of hybridizations in the study: ~ 10

5) A common reference RNA was not used.

6) Quality control measures were not used. No replicates were done. Dye swap was not used.

7) Description of the experiments:

a) To identify and analyze mtDNA mutation-responsive genes, a comparison of gastrocnemius muscle tissues from WT (control) and D257A mice was conducted. We examined changes in gene expression in the muscle associated with mtDNA mutations.

b) Currently, we are submitting a manuscript (Title: Mitochondrial DNA Mutations Induce Mitochondrial Dysfunction, Apoptosis and Sarcopenia in Skeletal Muscle of Mitochondrial DNA Mutator Mice) to *PLoS ONE*. Our microarray data will be linked to the publication.

*Samples used, extract preparation and labeling*:

1) Bio-source properties:

a) All mice were males.

b) Mice were divided into two groups: a 13 month-old WT group and a 13 month-old D257A group. Gastrocnemius muscle tissues were taken from these two groups.

c) The area of muscle tissue investigated was the gastrocnemius.

d) Strain: *Polg*^D257A/D257A^ mice. Homozygous knock-in mice expressing a proofreading-deficient version of the mitochondrial DNA polymerase gamma (*Polg*).

e) These knock-in mice display a number of age-related phenotypes by 9 months of age. For additional details about the strain used please refer to the following reference:

Kujoth, G.C. *et al*. (2005) Mitochondrial DNA mutations, oxidative stress, and apoptosis in mammalian aging. *Science* 309, 481-4.

2) Biomaterial manipulations:

a) All mice were raised under 12:12 dark/light conditions, and housed in the specific pathogen-free facility at University of Wisconsin-Madison, and provided a nonpurified diet and acidified water ad libitum.

3) Extract preparation:

a) Gastrocnemius muscle tissue was dissected and immediately flash-frozen in liquid nitrogen.

b) Total RNA was isolated from muscle tissues using the TriZol Reagent (Invitrogen) according to the manufacturer's instructions.

4) Labeling protocol: cDNA synthesis from total RNA (Invitrogen), cRNA amplification and labeling (Enzo BioArray IVT kit), hybridization (hybridization oven 640) (Affymetrix), and washing and staining (fluidics station 400) (Affymetrix) were accomplished according to the manufacturer’s instructions.

5) External controls: Affymetrix standard spike controls were used in all experiments (eukaryotic hybridization control kit).

For additional information on hybridization procedures and reagents please visit www.Affymetrix.com.

*Measurement data and specifications*:

1) Arrays were scanned on the GeneChip Scanner 3000 (Affymetrix).

2) Image analysis was performed using GCOS 1.3 (Affymetrix).

3) Normalized and summarized data:

a) Each array was scaled (all probe sets) to a target signal of 500 before comparison in

GCOS 1.3. Each experimental array was compared to its particular control array. To determine the effects of aging associated with mtDNA mutations, each WT sample (*n* = 5) was compared to each D257A sample (*n* = 5), generating a total of twenty-five pairwise comparisons. We performed Parametric Analysis of Gene Set Enrichment (PAGE) to identify classes of genes that were differentially expressed as a result of mtDNA mutations. Genes were annotated with functional data from the Gene Ontology (GO) consortium (http://www.geneontology.org). We considered GO terms that were annotated at Level 3 or greater and were represented by at least 10 but not more than 1000 genes. A GO term was considered to be significantly changed by treatment if the p-value for both the PAGE and a False Discovery Rate (FDR) analysis was < 0.01. We also calculated z ratios for each gene set, which serve as a normalization factor. Gene expression changes were called statistically significant when the *P* value was < 0.05 and FC was > 1.0 for identification of mtDNA mutations-induced genes. The complete set of microarray data has been submitted to the GEO (Gene Expression Omnibus) repository (GSE21189).

b) Summarized data: Please refer to Fig 2A-C and Supporting Table 1.
